# Conserved Residues Lys^57^ and Lys^401^ of Protein Disulfide Isomerase Maintain an Active Site Conformation for Optimal Activity: Implications for Post-Translational Regulation

**DOI:** 10.3389/fmolb.2018.00018

**Published:** 2018-02-28

**Authors:** Cody Caba, Hyder Ali Khan, Janeen Auld, Ryo Ushioda, Kazutaka Araki, Kazuhiro Nagata, Bulent Mutus

**Affiliations:** ^1^Department of Chemistry and Biochemistry, University of Windsor, Windsor, ON, Canada; ^2^Laboratory of Molecular and Cellular Biochemistry, Faculty of Life Sciences, Kyoto Sangyo University, Kyoto, Japan; ^3^Molecular Profiling Research Center for Drug Discovery, National Institute of Advanced Industrial Science and Technology, Tsukuba, Japan

**Keywords:** lysine acetylation, oxidative protein folding, enzyme kinetics, protein disulfide isomerase, redox, thiol-disulfide exchange

## Abstract

Despite its study since the 1960's, very little is known about the post-translational regulation of the multiple catalytic activities performed by protein disulfide isomerase (PDI), the primary protein folding catalyst of the cell. This work identifies a functional role for the highly conserved CxxC-flanking residues Lys^57^ and Lys^401^ of human PDI *in vitro*. Mutagenesis studies have revealed these residues as modulating the oxidoreductase activity of PDI in a pH-dependent manner. Non-conservative amino acid substitutions resulted in enzyme variants upwards of 7-fold less efficient. This attenuated activity was found to translate into a 2-fold reduction of the rate of electron shuttling between PDI and the intraluminal endoplasmic reticulum oxidase, ERO1α, suggesting a functional significance to oxidative protein folding. In light of this, the possibility of lysine acetylation at residues Lys^57^ and Lys^401^ was assessed by *in vitro* treatment using acetylsalicylic acid (aspirin). A total of 28 acetyllysine residues were identified, including acLys^57^ and acLys^401^. The kinetic behavior of the acetylated protein form nearly mimicked that obtained with a K57/401Q double substitution variant providing an indication that acetylation of the active site-flanking lysine residues can act to reversibly modulate PDI activity.

## Introduction

The pioneering work by independent researchers Anfinsen (Goldberger et al., 1964) and Straub (Venetianer and Straub, 1963, 1964, 1965) led to the discovery of a ubiquitous folding catalyst later regarded as protein disulfide isomerase (PDI: EC 5.3.4.1). This horseshoe-shaped enzyme is classified as a multifunctional thiol-disulfide oxidoreductase, comprised of four thioredoxin-like domains (*a*-*b*-*b*′-*a*′) and a polybasic C-terminal extension (*c*) (Kemmink et al., 1997; Noiva, 1999; Hatahet and Ruddock, 2009; Ali Khan and Mutus, 2014). The primary localization of PDI is within the endoplasmic reticulum (ER) where it functions as a foldase and chaperone in order to minimize the timescale during oxidative protein folding (Hudson et al., 2015). It is associated with NAD(P)H oxidase regulation (Janiszewski et al., 2005), thrombus formation (Kim et al., 2013) and nitric oxide (NO) transport from red blood cells to the endothelium tissue in an oxygen-dependent manner (Kallakunta et al., 2013; Ali Khan and Mutus, 2014), among many other roles. Involvement in such an array of cellular processes is indicative of a potential for activity regulation via reversible post-translational modifications. This is especially true when considering the ubiquitous nature of PDI, its long half-life and high expression levels at near 1% the total cellular protein content of some cell types (Goldberger et al., 1964; Root et al., 2004; Xu et al., 2014). Moreover, PDI has garnered much attention in view of its postulated roles in promoting pathogen internalization (Barbouche et al., 2003; Conant and Stephens, 2007), cancer (Benham, 2012), coagulation disorders (Jurk et al., 2011; Cho et al., 2012), and *S*-nitrosylated PDI being a mediator of protein aggregation in neuronal diseases including Alzheimer's, Parkinson's and Amyotrophic Lateral Sclerosis (Conway and Harris, 2015). As a result, there is a growing need to elucidate the mechanisms underlying the modulation of its multiple activities with the view of designing new therapeutics to specifically target this multifunctional enzyme.

**Figure 1:**
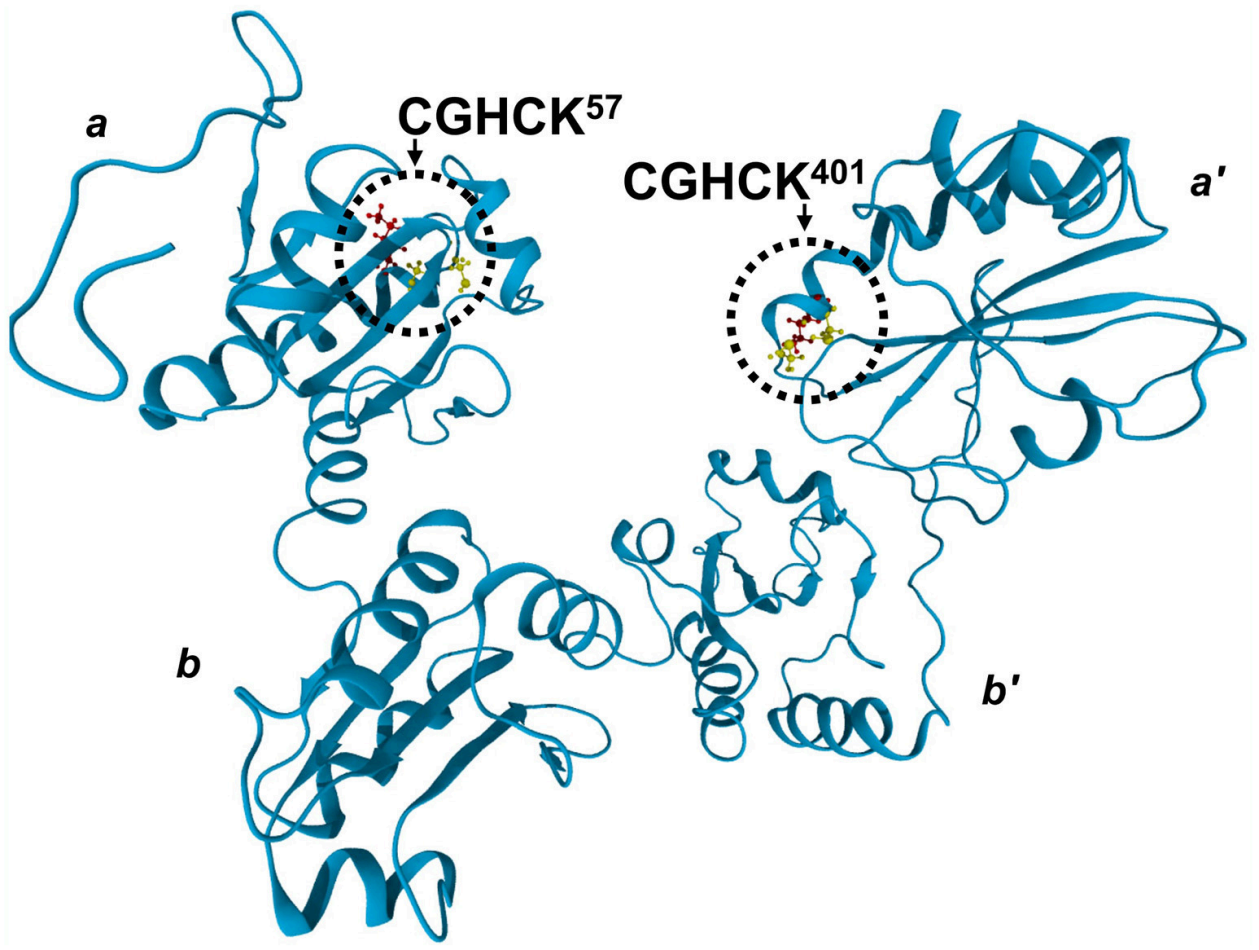
The crystal structure of reduced PDI in the reduced state (PDB: 4EKZ) (Wang et al., 2013). The *a-b-b*′*-a*′ thioredoxin-like domains are indicated, along with the conserved CGHCK active site motifs of the *a*-type domains. Ball and stick representation of the active site vicinal thiols (yellow) and the highly conserved neighboring lysine residues (red) is provided.

PDI has two catalytically active CxxC motifs, each located in domains *a* and *a*′ (Figure 1). The active sites reside on a flexible loop at the N-cap of an α-helix. These redox-active centers facilitate thiol-disulfide exchange and isomerization reactions and have similar reduction potentials (Chambers et al., 2010). The N-terminal cysteine (Cys_N_) has an anomalously low pKa in the range of 3.8–5.6 (Kortemme et al., 1996; Lappi et al., 2004; Karala et al., 2010), rendering it a potent nucleophile poised for the initial attack of a substrate's disulfide. The C-terminal active site cysteine (Cys_C_) of the vicinal thiol couple is the resolving cysteine. It functions to release PDI from a mixed-disulfide intermediate state (Walker et al., 1996; Lappi et al., 2004; Wilkinson and Gilbert, 2004). The intervening residues, denoted by x (where x is any amino acid), have been widely described for their influence on the reduction potentials and subsequent disulfide stabilities of the CxxC motif when oxidized (Woycechowsky and Raines, 2003). It is the importance of active site-flanking residues that remain ill-described. Human PDI (hPDI) contains a highly conserved active site sequence found among different PDI family members as being YAPWCGHCK (Kimura et al., 2004). A lysine residue (underlined) adjacent and directly C-terminal to the active site was shown to facilitate a significant enhancement of the rate of isomerization as determined using the insulin turbidity assay. Indeed, these findings would suggest a potential for modulating PDI activity via post-translational modifications. One such being *N*^ε^-lysine acetylation of the aforementioned conserved active site lysine residues.

Only recently has lysine acetylation been recognized for its importance with regards to non-histone and non-nuclear proteins (Smith and Workman, 2009). More importantly, ER-luminal lysine acetylation has been identified (Costantini et al., 2007), along with an acetyl-CoA transporter and two distinct ER-luminal transmembrane lysine acetyltransferase enzymes (Ko and Puglielli, 2009; Jonas et al., 2010). Proteomic assessment of purified ER preparations from human neuroglioma cells identified Lys^401^ of the *a*′-domain active site of hPDI as being acetylated (Pehar et al., 2012). Based on this, the goal of this study was to assess the importance of the highly conserved active site-flanking lysine residues Lys^57^ and Lys^401^ on the kinetics of hPDI activity. Non-conservative amino acid substitutions and chemical acetylation using acetylsalicylic acid, the active ingredient in the popular therapeutic aspirin, were performed. Enzyme kinetics were measured directly and continuously using dieosin glutathione disulfide (di-E-GSSG) (Raturi and Mutus, 2007), a previously designed pseudo-substrate that exploits the phenomenon of fluorescence self-quenching (FSQ). By monitoring the rate of disulfide bond cleavage (i.e., reduction of di-E-GSSG to two EGSH molecules) we were able to discern both the oxidoreductase and single-turnover thiol-reductase kinetics of wild type hPDI and the site-directed substitution variants in which Lys^57^ and Lys^401^ were substituted by glutamine, alanine, or glutamic acid. A particular focus of the analyses was placed on the glutamine substitution variants due to the prevalence of the CGHCQ motif in other PDI family members and homologs (instead of CGHCK). The significance of Lys^57^ and Lys^401^ to oxidative protein folding was assessed by monitoring the reaction between hPDI and the ER-resident oxidase, endoplasmic reticulum oxidoreductin-1α (ERO1α). The hPDI-ERO1α pathway is a pivotal step in “PDI-1^st^” oxidative protein folding (Hudson et al., 2015). Here, oxidized PDI (oxPDI) transfers oxidizing equivalents to reduced (nascent) protein substrates to introduce disulfide bonds and promote the native fold. In doing so PDI becomes reduced. The regeneration of oxPDI is achieved by ERO1α-mediated oxidation which follows an electron transfer pathway terminating with the reduction of molecular oxygen (O_2_) (Araki and Nagata, 2011; Araki et al., 2013). We show that intact CxxC-flanking lysine residues enhance oxidoreductase and thiol-reductase activities, as well as promote efficient electron transfer between hPDI and ERO1α. Herein, PDI will be used synonymously for hPDI. Amino acid numbering is based on the accepted sequence for PDI from *Homo sapiens* (Uniprot: PDIA1, P07237).

## Materials and methods

### Site directed mutagenesis

Substitution of residues Lys^57^ and Lys^401^ was performed using the Q5 site-directed mutagenesis kit (NEB) as per the manufacturer's instructions. Primers were designed end-to-end with the forward primer containing the mutagenic sequence that resulted in a single amino acid substitution to either alanine, glutamine, or glutamic acid. The polymerase chain reaction conditions were optimized for each primer set (Supplementary Table 1). The pET-28b derivatives containing mutagenized N- and C-terminally 6-His tagged PDI cDNA were sequenced by Robart's Research Institute (London Regional Genomics Center, London, Ontario, Canada). A total of nine PDI variants were designed: K57Q, K57A, K57E, K401Q, K401A, K401E, K57/401Q, K57/401A, and K57/401E. Additionally, active site cysteine mutations were engineered for control experiments following the same protocol (see below).

### Purification of PDI

*Escherichia coli* BL21 (DE3) cells (NEB) expressing the PDI variant of interest were grown in 1.5 L of fresh 2 × YT media containing 50 μg/ml kanamycin sulfate. At a cell density (OD_600_) of 0.6 overexpression was induced using 1 mM IPTG with incubation for 4 h at 37^O^C with vigorous shaking. Cells were harvested by centrifugation and resuspended in 30 ml of homogenization buffer (50 mM Tris-HCl pH 8.0, 1 mM NaCl, 1% Triton X-100, 2 mM PMSF, 125 μg/ml lysozyme, and 75 μg/ml DNase) followed by 10 rounds of sonication on ice at 20 s pulses (Sonic Dismemberator, Fisher Scientific). Cellular lysates were clarified by centrifugation at 12,000 × g for 30 min and passed over a gravity-fed HIS-Select Ni-affinity resin (Sigma) with a 5 ml bed volume. The affinity resin was then washed with 3 column volumes of wash buffer (50 mM potassium phosphate pH 8, 150 mM NaCl) containing 10 mM imidazole, followed by another wash using 2 column volumes of wash buffer containing 40 mM imidazole. Recombinant PDI was eluted using a 500 mM imidazole wash buffer. The eluate was incubated with 100 mM dithiothreitol (DTT) for 20 min on ice, then desalted and buffer exchanged with PDI assay buffer (0.1 M potassium phosphate pH 7.4, 0.1 mM diethylenetriaminepentaacetic acid) using Amicon Ultra-15 centrifugal filter units (MWCO: 30 kDa) as per the manufacturer's instructions (EMD). Protein concentration was determined using the BCA assay (Smith et al., 1985), and purity was assessed via SDS-PAGE (Supplementary Figure 1A). This procedure yielded a total of between 36 and 50 mg of pure protein. Enzyme preparations were diluted to 1 mg/ml in PDI assay buffer, aliquoted, snap-frozen in liquid nitrogen, and stored at −80^O^C.

For all enzyme preparations the functional concentrations were determined using the di-E-GSSG fluorogenic assay (Raturi and Mutus, 2007). Briefly, 10 μl of affinity purified PDI was incubated with 800 nM di-E-GSSG in PDI assay buffer and allowed to react for 20 min, or until maximum fluorescence was reached. This provided an indication that all catalytically active enzyme had reduced di-E-GSSG to EGSH. The resulting maximum fluorescence was plotted against a standard curve to determine the concentration of active PDI on a per-active site basis (refer to Supplementary Figure 2). This allowed for the correction of any differences in the apparent protein quantities observed among the purifications of each variant. A standard curve was generated by fully reducing known concentrations of di-E-GSSG (25–800 nM) with 1 M DTT. Fluorescence values were then related to the concentration of the EGSH product. In-turn, the fluorescence generated by the PDI-catalyzed reduction of di-E-GSSG was translated to a known concentration of EGSH. The concentration of functional PDI (active sites) was obtained by considering the formation of 2 EGSH molecules per active site.

### Purification of ERO1α

In this study, a 6-His tagged constitutively active human ERO1α variant (C104/131A) was used (Araki and Nagata, 2011). Purification followed an adapted protocol from both Araki and Nagata (2011) and that of the PDI purification procedure described. *E. coli* BL21 (DE3) pLysS cells (NEB) containing the 6-His tagged ERO1α construct were grown overnight in 1.5 L of fresh 2 × YT media containing ampicillin and chloramphenicol antibiotics (100 and 25 μg/ml, respectively) and 10 μM flavin adenine dinucleotide (FAD). As FAD is photo-labile, the culture was protected from light at all times. At an OD_600_ of 1.0, ERO1α expression was induced using 0.5 mM IPTG for 16 h at room temperature. Cells were harvested, lysed in the presence of 5 μM FAD and ERO1α was isolated and purified as per the methods described previously for PDI. Affinity purified ERO1α was completely oxidized using 20 mM potassium ferricyanide (KFe(CN)_6_) for 20 min on ice. After desalting and buffer exchange, the isolated enzyme was stored at −80^O^C in storage buffer (20 mM HEPES sodium salt pH 7.4, 150 mM NaCl, 10% glycerol) at a concentration of 2 mg/ml as determined using the BCA assay. The purified holoenzyme (FAD-bound ERO1α) exhibits a characteristic absorbance peak at 440 nm (ε = 12.5 mM^−1^ cm^−1^; Inaba et al., 2010). Taken in conjunction with the total protein concentration determined using the BCA assay, the purity of the FAD-bound enzyme was > 92%. SDS-PAGE was also performed (Supplementary Figure 3).

### *In vitro* kinetics

All kinetic assays described were performed at 22 ± 2°C using enzyme having gone through no more than one freeze-thaw cycle. Furthermore, each was performed in parallel with wild type or an untreated control. Data are reported as the mean and standard deviation (±*SD*) of *n* technical replicates. All fluorescence data were collected using a Cary Eclipse fluorescence spectrophotometer (5 nm slit width and medium gain settings; Agilent Technologies) unless otherwise stated.

#### Steady-state oxidoreductase di-E-GSSG assay

The fluorescent di-E-GSSG pseudo-substrate was synthesized as previously described (Raturi and Mutus, 2007). The assay used to measure the oxidoreductase activity of PDI was performed in PDI assay buffer of varying pH at a final volume of 500 μl. PDI (10 nM) was added to a cuvette containing 10 μM DTT and varying concentrations of di-E-GSSG (25 nM to 5 μM). The reduction of di-E-GSSG was monitored for 60 s and the initial rate of EGSH production was determined from the linear portion of the curve (RFU vs. time; λ_ex_: 525 nm, λ_em_: 545 nm). Initial velocity values were fit to a Michaelis-Menten hyperbolic curve (Equation 1) using non-linear regression analysis (Brown, 2001) in Excel with the Solver data analysis tool.

(1) $$\begin{array}{r} v_{o}=\frac{V_{\max}\left[S\right]}{K_{M}+\left[S\right]} \end{array}$$

pH-dependence experiments were performed under steady-state conditions using a single di-E-GSSG concentration of 800 nM. The data were fit to a double-ionization model providing a bell-shaped curve (Equation 2). Where v represents the rate of fluorescence increase at any given pH.

(2) $$\begin{array}{r} v_{o}=\frac{10^{pH-{pK}_{1}}}{\left(1+10^{pH-{pK}_{1}}\right)}-\frac{10^{pH-{pK}_{2}}}{\left(1+10^{pH-{pK}_{2}}\right)} \end{array}$$

#### Single turnover kinetic studies

The single-turnover rate of PDI was measured using the di-E-GSSG assay where 80 nM of PDI was incubated with 1 μM di-E-GSSG in the absence of DTT. The reaction was monitored until completion at varying pH. A time-course fluorescence increase was related to the consumption of di-E-GSSG over time, resulting in decay plots (Supplementary Figure 8) which were fit to Equation 3 (refer to Supplementary Figure 8).

(3) $$\begin{array}{r} {\left[di-E-GSSG\right]}_{f}={\left[di-E-GSSG\right]}_{o}e^{-k_{obs}t} \end{array}$$

Where [di-E-GSSG]_f_ and [di-E-GSSG]_o_ represent the final and initial concentrations of the substrate over the course of the reaction, respectively. The observable rate, *k*_obs_, was taken as the slope of the natural log of the integrated rate law above (Equation 3).

#### ERO1α oxidase assay

ERO1α oxidase activity was measured using an oxygen consumption assay monitored by an Oxygraph Plus Clark-type oxygen electrode (Hansatech) with a stirrer speed of 70%. Experiments were performed in air-saturated ERO1α assay buffer (50 mM HEPES sodium salt pH 7.4, 150 mM NaCl, 2 mM EDTA) at a final reaction volume of 800 μl. Air-saturation of the buffer was completed by bubbling with air for 30 min prior to each experiment. A mixture containing 5 μM PDI and 20 mM GSH was incubated for 1 min prior to the addition of 2 μM ERO1α. Reactions were performed in the dark to prevent the photo-degradation of the FAD coenzyme. The initial linear portion of the curves were used to report observable rates as determined by the magnitude of the slope of the best-fit lines (*R*^2^ ≥ 0.99).

### Aspirin-mediated acetylation of PDI

*N*^ε^-lysine acetylation of PDI was carried out using acetylsalicylic acid (ASA). The reaction was performed in a 37°C oven for 4 h in a 0.1 M Tris-HCl buffer of pH 8.5 using 0.1 to 15 mM ASA with 0.5 mM DTT. Hydroxylamine (HA) was then added to quench the acetylation of primary amines and to revert undesirable *O*-acetylation, (Münchbach et al., 2000; Pflieger et al., 2008; Kadiyala et al., 2012; Wiktorowicz et al., 2012; Hatimy et al., 2015; Meert et al., 2016). HA-mediated acyl removal was accomplished by adding 100 mM HA to the acetylation mixture and allowing it to react for 30 min at room temperature. Lysine acetylation was confirmed by Western blot analysis and ultra-high-performance liquid chromatography electrospray ionization mass spectrometry (refer to Supplementary Methods).

### Statistical analysis

One-way ANOVA followed by Dunnett's multiple comparisons test was performed on all kinetic data using GraphPad Prism version 6.0e (GraphPad Software, La Jolla California, USA). Differences were deemed significant if *P* < 0.05. Significance is denoted with respect to wild type control unless otherwise stated; *P < 0.05, **P < 0.01, ***P < 0.005, ****P < 0.001. No significance is denoted by *ns* where appropriate.

## Results and discussion

### Substitution of Lys^57^ and Lys^401^ attenuates PDI oxidoreductase and thiol-reductase activity

Characterization of the conserved residues important and crucial for catalysis has helped to elucidate details of the PDI oxidoreductase mechanism (Kortemme et al., 1996; Dyson et al., 1997; Lappi et al., 2004; Roos et al., 2007, 2009, 2010; Hernández et al., 2008). Conflicting reports have identified an active site-flanking lysine as being a requirement for optimal catalytic function. The insulin reduction and RNase A refolding assays revealed that substituting the active site-flanking Lys (CGHCK) with Arg (CGHCR) in the first domain active site of rat PDI led to a 15% loss of activity (Lu et al., 1992). Later, the hPDI family member PDIA6, regarded as hP5, was studied. This member has two CxxC motifs, the first is CGHCQ and the second is CGHCK. Interestingly, it was demonstrated that a Q-to-K substitution in the first active site resulted in a 45% increase of isomerase activity (Kimura et al., 2004). We have expanded on these previous findings.

In order to assess the potential catalytic contributions of Lys^57^ and Lys^401^ to full-length PDI, the pH-dependent activity profiles of the wild type and double-substitution variants (where both of the aforementioned lysine residues are substituted) were determined using the di-E-GSSG assay. The structural integrity of PDI and the fluorescent properties of the di-E-GSSG substrate were first determined over a comprehensive range of pH. For PDI, the intrinsic tryptophan fluorescence spectrum was monitored as a simple reporter of pH-induced conformational changes. Spectra remained stable and consistent over the pH range of 5.5–9.5. However, the emission peak began to increase at pH 4.5, indicating that the enzyme was undergoing substantial global conformational changes (Supplementary Figure 4A). The stability of di-E-GSSG within the pH range of 5.5–9.5 was also tested by completely reducing the GSSG disulfide with excess DTT. The substrate gave a consistent 56-fold fluorescence-enhancement upon disulfide reduction irrespective of pH (Supplementary Figure 4B).

**Figure 2:**
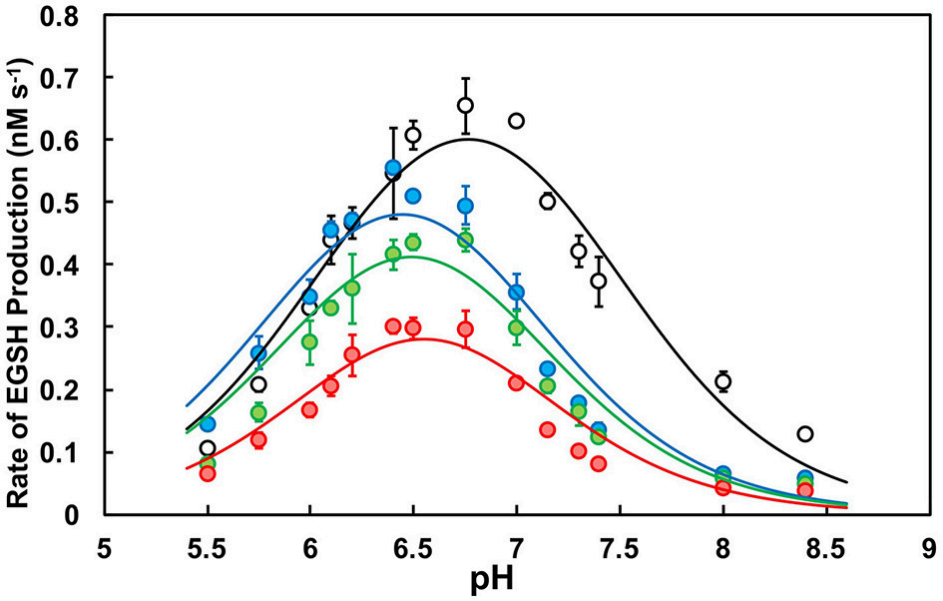
pH-dependence of the oxidoreductase activity of PDI. Activity profiles of wild type (*WT*; black), K57/401Q (blue), K57/401A (green), and K57/401E (red). Ten nanomolar PDI was incubated with a fixed concentration of di-E-GSSG (800 nM) in PDI assay buffer of varying pH. The initial rate of product (EGSH) formation was monitored for the first 60 s of the reaction. pH-dependent activity profiles represent the mean ± *SD* of three experiments fit to Equation (2).

To our knowledge, we present for the first time a full *in vitro* pH-dependent activity profile for PDI over a comprehensive range. Assessment of the initial rates of reaction in relation to pH at a fixed concentration of substrate (di-E-GSSG) showed that all PDI enzymes exhibited bell-shaped profiles with narrow breadths. This suggests that the optimal conditions for functioning as an oxidoreductase occurs over a small range of pH (Figure 2). Fitting the data to a double-ionization model (Equation 2) allowed for the estimation of two macroscopic p*K*_a′_s: ^app^p*K*_1_ and ^app^p*K*_2_. The ascending limb of the fits represents enzyme activation as a result of an increasing fraction of thiolate anion (R-S^−^) in relation to the thiol-form (R-SH) of the active site cysteines. The macroscopic p*K*_a_ determinations make it difficult to assign, unambiguously, the ionization of particular residues, including distinguishing between Cys_N_ and Cys_C_; or the active sites themselves. We interpret ^app^p*K*_1_ as being a likely indicator of the combined contribution from the ionization of Cys_C_ in each active site. This notion is supported by previous studies identifying Cys_C_ as having a p*K*_a_ in the range of 6.1, while Cys_N_ is considerably lower (Kortemme et al., 1996; Lappi et al., 2004; Karala et al., 2010). This would suggest that *(1)* at low pH Cys_C_ is responsible for rate limitation and *(2)* each active site exhibits similar reactivity.

To explore *(1)* the possibility of Cys_C_ being the limiting factor in PDI activity at low pH, a substrate-trapping experiment was designed to test its effectiveness in resolving PDI from a mixed-disulfide state. As illustrated in Supplementary Figure 5, under our steady-state conditions, at acidic pH (5.5) PDI exhibited zero-order kinetics with respect to varying di-E-GSSG concentration (Supplementary Figures 5A,B). An investigation of this indicated that ~92% of wild type PDI active sites were trapped in a mixed-disulfide state at pH 5.5, compared to only ~30% at pH 7.4 (Supplementary Methods; Supplementary Figure 5C). Thus, at low pH, the stabilized mixed-disulfide state points to a marked attenuation of substrate release as mediated by Cys_C_. Coupled with our findings for ^app^p*K*_1_ it is likely that at this low pH there is a substantially smaller proportion of the reactive Cys_C_ thiolate species in relation to the cysteinyl thiol (pH < p*K*_a_), thereby accounting for more trapped PDI molecules (Walker and Gilbert, 1997; Roos et al., 2009).

The idea that *(2)* the active sites are very similar has been shown to be true from a thermodynamics stand-point, where each displays similar reduction potentials (Chambers et al., 2010). This is despite having dissimilar orientations about the PDI architecture (Wang et al., 2013; Xu et al., 2014). In light of this, the cysteine p*K*_a′_s of the *a* and *a*′-domain active sites can be estimated as being quite similar. To expand on this, we generated active site variants whereby the vicinal thiol couple was substituted to alanine, CxxC-to-AxxA, either individually or simultaneously. Our *in vitro* results showed that each active site does indeed function nearly identically under the conditions employed using the di-E-GSSG substrate (Supplementary Figure 6). Substitution of all four redox active cysteine residues unsurprisingly resulted in an enzyme variant with no activity. Single active site CxxC-to-AxxA substitutions to the *a* and *a*′-domains generated variants 57 and 50% as active as wild type, respectively (Supplementary Figure 6A).

All enzyme variants tested were determined to have similar ^app^p*K*_1_ values between 6.05 and 6.2 (Table 1). It is apparent that the catalytic contributions of Lys^57^ and Lys^401^ do not affect enzyme activation (see above). However, the pH at maximum activity (pH_max_) as well as the maximum rate of product (EGSH) formation were affected by the simultaneous substitutions. The double-substitution glutamine, alanine, and glutamic acid variants produced activities at pH_max_ that were ~68, 50, and 38% of the wild type enzyme, respectively. The activity decrease was more dramatic at physiological pH 7.4, where the glutamine, alanine, and glutamic acid double substitution variants displayed rates of di-E-GSSG reduction that were ~36, 33, and 21% of the wild type enzyme, respectively (Figure 2). When catalyzing disulfide reduction between pH 7.0 and 7.4 wild type PDI outperformed the double-substitution variants to the greatest extent. This coincides closely to the ER luminal environment, meaning that not only does substitution to the active site lysine residues attenuate activity, but the perturbation of the pH-dependence is likely significant with regards to the subcellular localization of PDI (Wu et al., 2000; Casey et al., 2010).

**Table 1:** Apparent p*K*_a_ values and the pH of maximum activity (pH_max_) of wild type (*WT*) and the double substitution PDI variants.

**PDI**	**^app^pK_1_**	**^app^pK_2_**	**pH_max_**
WT	6.16 ± 0.01	7.37 ± 0.02	6.77 ± 0.16
K57/401Q	6.05 ± 0.01^**^	6.90 ± 0.00^****^	6.44 ± 0.00^**^
K57/401A	6.11 ± 0.03^*^	6.87 ± 0.04^****^	6.49 ± 0.04^**^
K57/401E	6.20 ± 0.01	6.80 ± 0.01^****^	6.55 ± 0.01^*^

*Data were extracted from the fits seen in Figure 2 and represent the mean ± SD of three experiments*.

* P < 0.05,

** P < 0.01,

**** *P < 0.001*.

The most obvious effect of double-substitution was ^app^p*K*_2_ being shifted to the left by ~0.5 pH units (Table 1). The net result of this was the shift of pH_max_ by ~0.3 pH units in comparison to wild type (Figure 2; Table 1). This provided an indication that ^app^pK_2_, the descending limb representing enzyme inactivation, was directly dependent on residues at position 57 and 401. Due to the shift being nearly constant among the differing PDI variants, we suggest that a lysine adjacent the active site provides the *specific* physicochemical character to elicit the pH-dependence of activity.

**Figure 3:**
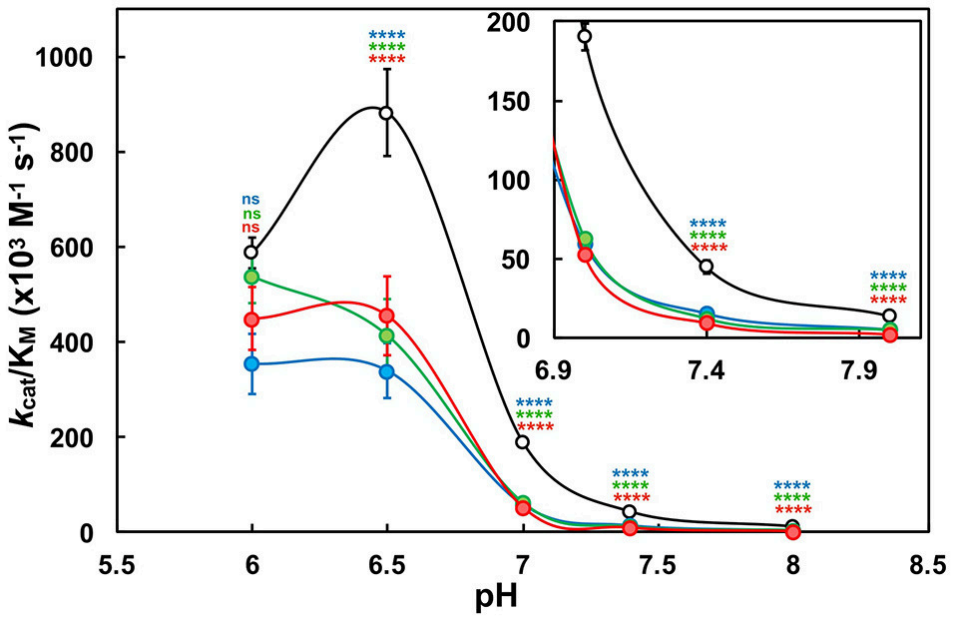
The pH-dependence of oxidoreductase efficiency (*k*_cat_/K_M_) under steady-state conditions. Wild type PDI (*WT*; black) and double-substitution variants (K57/401Q, blue; K57/401A, green; K57/401E, red) at a concentration of 10 nM were incubated with the di-E-GSSG pseudo-substrate (25 nM to 5 μM) in the presence of 10 μM DTT to provide initial rates of substrate reduction. Statistical significance with respect to wild type is denoted by appropriately colored asterisks (^*^). Data reported as the mean ± *SD* of three experiments. The apparent fit to the data is qualitative and provided as a simple comparator between the protein forms.

First, the pH-dependence of the kinetic parameters: turnover number (*k*_cat_), Michaelis-Menten constant (K_M_) and catalytic efficiency (*k*_cat_/K_M_) were determined in the pH range of 6.0–8.0 to further elucidate a mechanistic contribution of the CxxC-flanking lysine residues (Supplementary Tables 2–6). Kinetic data below pH 6.0 were unattainable for reasons previously described (refer to Supplementary Figure 5). Enzyme efficiency was greatly attenuated as a result of substituting either or both Lys^57^ and Lys^401^. Overall, K57/401Q was ~3-fold less efficient than wild type, and K57/401A and K57/401E were upwards of 4- and 7-fold less efficient, respectively (Figure 3; Supplementary Tables 2–6). Although, at pH 6.0 there was no significant difference determined between wild type and the PDI variants; an indication of substrate turnover being largely influenced by poor Cys_C_ reactivity at low pH. Above pH 6.0, K_M_ and *k*_cat_ respectively increased and decreased, with the effect being exacerbated by the double substitution variants. This result suggests that an active site lysine mediates both optimal di-E-GSSG binding to the active site as well as enhanced catalytic turnover. Moreover, the wild type enzyme deviated from the substitution variants and outperformed them to the greatest extent within the physiologically relevant range of pH.

In an attempt to explain the apparently diminished substrate binding and turnover capabilities of mutant PDI, as well as the perturbations to ^app^p*K*_2_ (Figure 2), we sought to examine the structural aspects of related PDI family members naturally expressing a CGHC(K/Q) active site motif. The focus here was on NMR solution structures because these provide a dynamic view of protein conformations. Interestingly, by analyzing the solution structures of homologous mammalian PDI family members PDIA3 (PDB: 2DMM) and PDIA6 (PDB: 2DMM) strong hints as to the potential role of Lys^57^ and Lys^401^ were observed. The PDB entry 2DMM contains 20 solution structures of a 142-amino acid peptide from the *a*′-domain of human PDIA3; naturally harboring a CGHCK active site motif (akin to wild type PDI, PDIA1). Whereas the PDB entry 2DML represents 20 solution structures of a 130-amino acid peptide from the *a-*domain of mouse PDIA6 which contains a CGHCQ active site sequence (akin to the K-to-Q PDI variants designed here).

**Figure 4:**
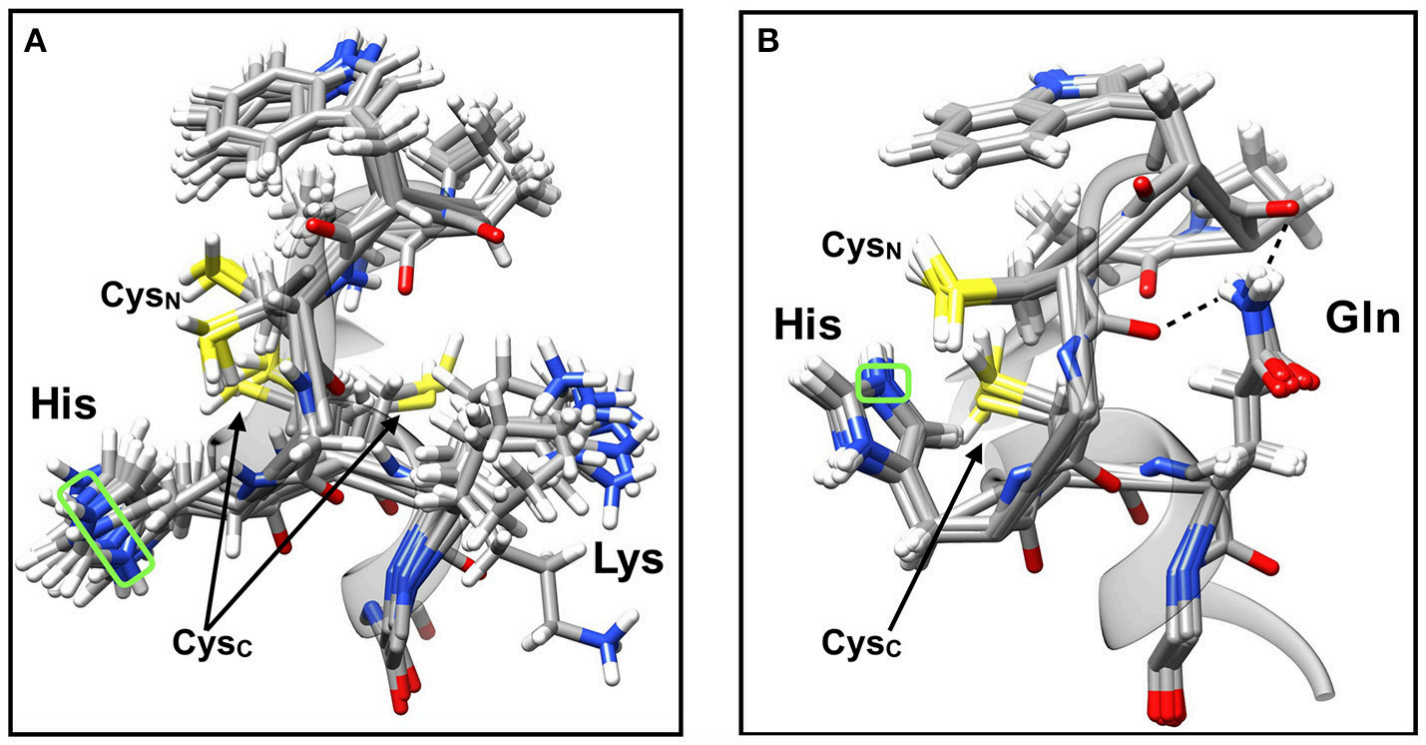
NMR solution structures of the active site CGHCK and CGHCQ motifs naturally found in the PDI family members PDIA3 and PDIA6, respectively. **(A)** Superimposition of 20 solution structures of the *a*′-domain active site of PDIA3 (APWCGHCK), PDB: 2DMM. **(B)** Superimposition of 20 solution structures of the *a*-domain active site of PDIA6 (APWCGHCQ), PDB: 2DML. Hydrogen bonding is represented by dotted lines. The N_ε_ atom of the intervening histidine residue (boxed in green) was used to provide distance measurements with the sulfur of the Cys_N_ and Cys_C_ side chains. Visualized using UCSF Chimera software (Pettersen et al., 2004).

A comparison of the overlaid structures of 2DMM (CGHCK; Figure 4A) to that of 2DML (CGHCQ; Figure 4B) shows that when lysine is the C-terminal amino acid to the CGHC motif, the residue's side chain is very mobile with no true interactions with other neighboring atoms. This view is confirmed by previous reports demonstrating the short-lived, transient nature of backbone hydrogen bonds with lysine side chains (Zandarashvili et al., 2011). The result is in an apparently flexible active site loop. It is believed that the motion of the lysine side chain is translated through the conserved active site region to the intervening histidine, causing it to also be mobile and maintain a certain distance from the vicinal active site cysteines. The minimum apparent distance was determined to be between 5.2 and 7.8 Å to the sulfur of Cys_N_ and 6.0–11 Å to the sulfur of Cys_C_. Measurements were taken from the imidazolium N_ε_ atom based on the tautomeric state of the protonated free amino acid (Reynolds et al., 1973; Hansen and Kay, 2014). This shows the appreciable dynamics of the wild type active site and provides an indication that the intervening histidine is in closer proximity to Cys_N_ than Cys_C_. Full or partial positive charges <6.5 Å away are able to stabilize thiolate anions (Britto et al., 2002; Hernández et al., 2008). The distances measured here are supportive of histidine acting as a stabilizing factor for the Cys_N_ thiolate and not Cys_C_ (Kortemme et al., 1996; Hernández et al., 2008).

In contrast to the structure observed for 2DMM (CGHCK), when lysine is replaced with glutamine as in 2DML (CGHCQ), the δ-amide group of the side chain is immobilized by hydrogen bonding interactions with the backbone oxygen of the upstream active site cysteine (*n*−4) and proline (*n*−6) residues (FYAPWCGHCQ). As a result, the active site is apparently more ordered (Figure 4B). Backbone hydrogen bonding by the glutamine side chain is a well characterized feature of many protein motifs (Vijayakumar et al., 1999; Eswar and Ramakrishnan, 2000; Vasudev et al., 2012). As a result, the imidazolium moiety of the intervening histidine is apparently restricted in its motion and in much closer proximity to Cys_N_. The effect of this conformational change is 2-fold, where histidine causes a decrease of thiol p*K*_a_ as well as a steric impedance to the active site. This means that a CGHCQ motif would exhibit lower substrate affinity and attenuated turnover rates; that which has been observed with steady-state kinetic analyses (Figure 3). Measurements taken from the solution structure overlays indicate that the imidazolium N_ε_ is about 2.6 and 4.1 Å from the Cys_N_ and Cys_C_ sulfurs, respectively (Figure 4B). This again indicates that the intervening histidine is in closer proximity to Cys_N_. Compared to the CGHCK motif, it is at least ~3 Å closer to Cys_N_. As such, unfavorably strong ion-pairing likely results from this and the over-stabilization of the Cys_N_ thiolate could actually decrease its nucleophilicity, rendering it less reactive (Bulaj et al., 1998; Nagy, 2013). The invariable value determined for ^app^p*K*_1_ (the ionization of Cys_C_, Figure 2) among wild type PDI and the double-substitution variants shows that the proximity of histidine to Cys_C_ does not impose any observable effects to the residue. It is apparent that Cys_N_ is predominantly impacted by the conformational change of the active site.

The apparent flexibility of the active site may lend valuable insight as to the key characteristics of the CxxC motif. PDI has been described as promiscuous with few bona fide substrates (Appenzeller-Herzog and Ellgaard, 2008), although recent research is beginning to identify some degree of substrate specificity (Stopa et al., 2017). The active site of PDI is not so reliant on the classical lock-and-key fit with its substrates. The catalytic activity depends on thiol-disulfide accessibility and thermodynamic characteristics such as disulfide reduction potential and thiol p*K*_a_. We propose that a wild type active site allows for more freedom of movement, particularly with regards to the intervening histidine and catalytic cysteine residues. This provides the framework by which PDI can gain the necessary conformation of its active site to perform productive catalysis. We are presently pursuing this theory by performing complex molecular dynamics simulations of full-length PDI in both reduced and oxidized states coupled with substrate docking. It is well understood that PDI is a highly dynamic enzyme on a global scale (Wang et al., 2013). Recent examples of cooperativity among its domains (Bekendam et al., 2016) and substrate-specific effects to activity (Roth, 1981; Xu et al., 2014) have added to the need for more detailed structural studies about the active site locale. As such, it will be possible to identify with greater detail the residue-specific interactions that occur with regards to the marked shift to ^app^p*K*_2_ as centered on the substitution of Lys^57^ and Lys^401^. Whether the role an active site-flanking lysine plays in modulating ^app^p*K*_2_ occurs in the free enzyme (reduced or oxidized), the enzyme-substrate encounter complex or the mixed-disulfide state needs to be addressed.

**Figure 5:**
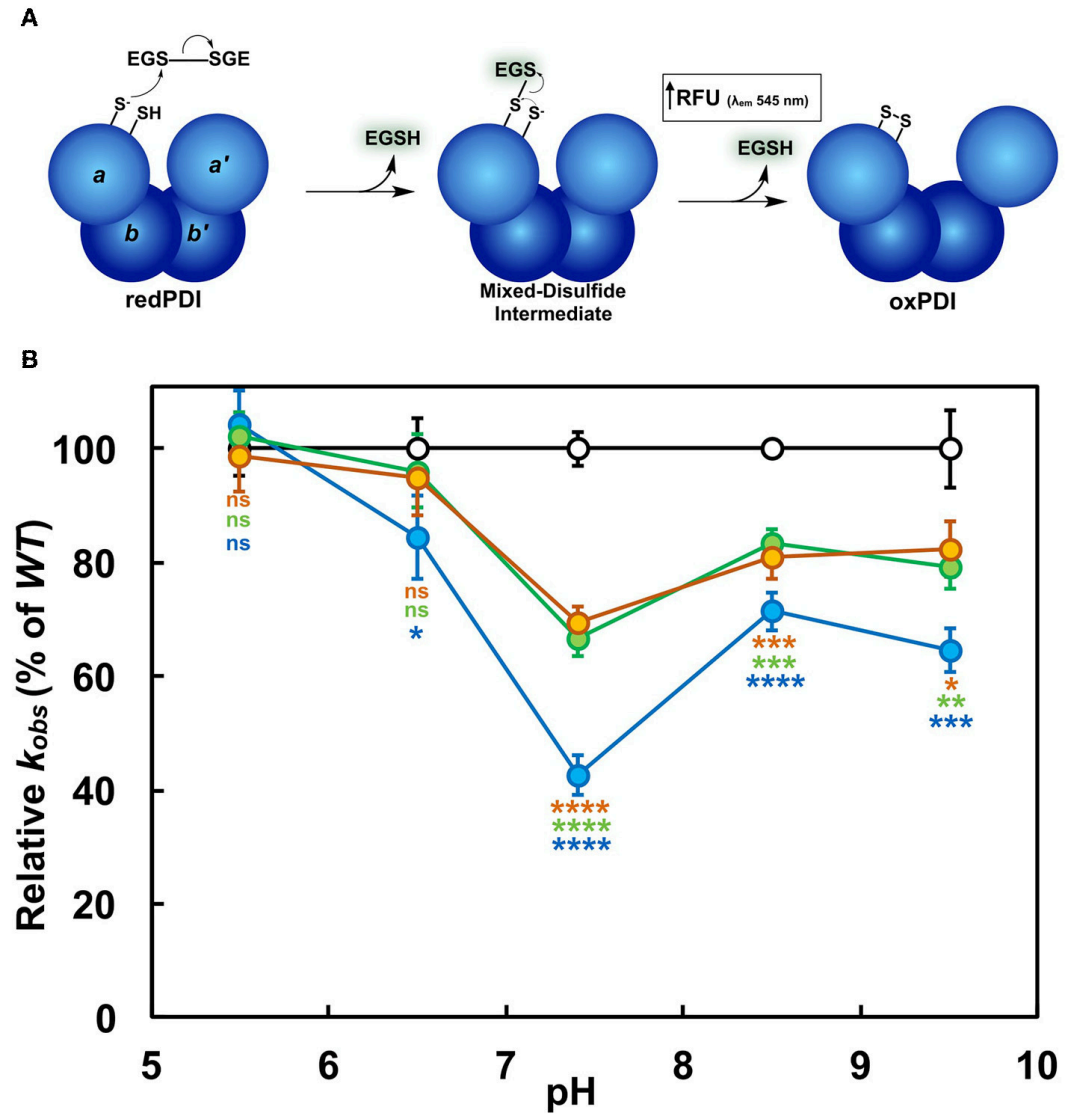
Single turnover thiol-reductase activity of PDI. **(A)** Schematic of PDI-catalyzed reduction of di-E-GSSG. Cys_N_ of redPDI initiates the reaction by nucleophilic attack of the GSSG disulfide, resulting in the formation of one free EGSH molecule and a mixed disulfide intermediate with a second EGSH molecule. The Cys_C_ resolving cysteine liberates PDI from the mixed-disulfide state. Di-E-GSSG reduction relinquishes FSQ, resulting in a detectable increase of fluorescence attributed to two free EGSH molecules per active site. For simplicity only the *a-*domain active site is depicted. **(B)** The relative observable rates (*k*_obs_) of K-to-Q PDI mutants (K57/401Q, blue; K57Q, green; K401Q, orange) were plotted as a percentage of wild type (*WT*; black). Statistical significance with respect to wild type is denoted by appropriately colored asterisks (^*^). Kinetic data represent the mean ± *SD* of three experiments.

The NMR structural studies show how Lys^57^ and Lys^401^ affect the balance between cysteine p*K*_a_ and nucleophilicity. A CxxC-flanking lysine residue thus functions to maintain an active site conformation that supports the optimal nucleophilicity of Cys_N_, as well as accessibility to the active site as a whole. We tested this by performing single turnover kinetics. The *in vitro* di-E-GSSG assay generally includes excess reducing agent, such as DTT herein, to facilitate a steady-state oxidoreductase catalytic cycle via the regeneration of reduced PDI active sites. This occurs without any contribution to an uncatalyzed rate of di-E-GSSG reduction. However, in the absence of DTT, each PDI molecule is capable of only a single turnover per active site, thereby providing a true measure of thiol-reductase kinetics (Figure 5A). It is important to note that once PDI catalyzes the reduction of di-E-GSSG, FSQ of the eosin moieties is abolished. The fluorescence generated is the result of two product EGSH molecules per active site. Even though one EGSH molecule forms a mixed-disulfide per PDI active site, its fluorescence is still detectable (refer to Supplementary Figure 5C). Thus, enzyme-catalyzed fluorescence increase is a single-phase event. A focus was placed on the K-to-Q variants here. As expected, at pH 5.5 the *k*_*obs*_ determined for each enzyme was unchanged. At pH 7.4 *k*_*obs*_ for K57/401Q was 2.5-fold less than that of wild type; the single domain K57Q and K401Q variants showed about 1.5-fold less activity than wild type (Figure 5B). With reference to the K57/401Q double substitution variant, a significant decrease in *k*_*obs*_ was also seen at pH 6.5, but not as much so at the more basic conditions tested. It should be noted that wild type PDI outperformed the mutants to the greatest extent at physiological pH, similar to the trend observed when catalyzing di-E-GSSG reduction under steady-state conditions (i.e., in the presence of DTT). Furthermore, it follows that no significant differences were observed between the respective single mutants. It is clear that the initiation of catalysis as mediated by Cys_N_ relies on the presence of an active site-flanking lysine. Substitution of such attenuates the rate of the initial nucleophilic attack of a substrate disulfide. This further corroborates the previous solution structure analyses which indicated the intervening histidine as negatively affecting Cys_N_ in the mutant (CGHCQ), but not the wild type (CGHCK) active site motif (Figure 4).

The domain organization of PDI proteins is believed to be indicative of the specialization each has with certain substrates (Hudson et al., 2015). As well, the role for CxxC-intervening residues in directly influencing the redox potential of thioredoxin-like enzymes allows for the fine-tuning of potent thiol-oxidase, thiol-reductase, or intermediate characteristics (Zapun et al., 1993; Chambers et al., 2010). Alongside this, our data provide for speculation as to the importance an active site-flanking lysine residue may have in defining certain characteristics crucial to PDI. The presence or absence of such may be a highly important distinguishing feature of the various PDI family members. Some orthologs of PDI, such as PDIL1-1, PDIL1-2, and PDIL1-4 found in *Arabidopsis thaliana*, naturally express a CGHCQ motif at one or each active site (Yuen et al., 2013). The differences highlight evolutionary variations that pose an example of how an active site-flanking lysine is tailored toward a more complex biological system. Therefore, our data suggest mammals harbor a more efficient oxidoreductase.

### CxxC-flanking lysine residues of PDI facilitate electron shuttling with ERO1α

The ability of PDI to reduce (or be oxidized by) ERO1α is a hallmark of oxidative protein folding. It has been previously shown that ERO1α functions to oxidize the *a*′-domain of PDI in order to restore the fully oxidized form of the enzyme. This being for the purposes of then introducing oxidizing equivalents to nascent polypeptides within the ER lumen (Araki and Nagata, 2011). The transfer of disulfides begins with activated ERO1α. PDI accepts these disulfides and in turn transfers oxidizing equivalents to some reduced substrate. Resultantly, electron flow occurs in the opposite direction, from said (reduced) substrate to PDI (first the *a*-domain then *a*′-domain), then to ERO1α where an intramolecular electron transfer occurs between the Cys^94^-Cys^99^ shuttle disulfide and the Cys^394^-Cys^397^ active site disulfide. The active site CxxC of ERO1α is proximal an FAD coenzyme, thus, when reduced, electron transfer generates FADH_2_ which in turn reduces O_2_ to form hydrogen peroxide (H_2_O_2_) (Baker et al., 2008; Inaba et al., 2010) (refer to Supplementary Figure 9).

**Figure 6:**
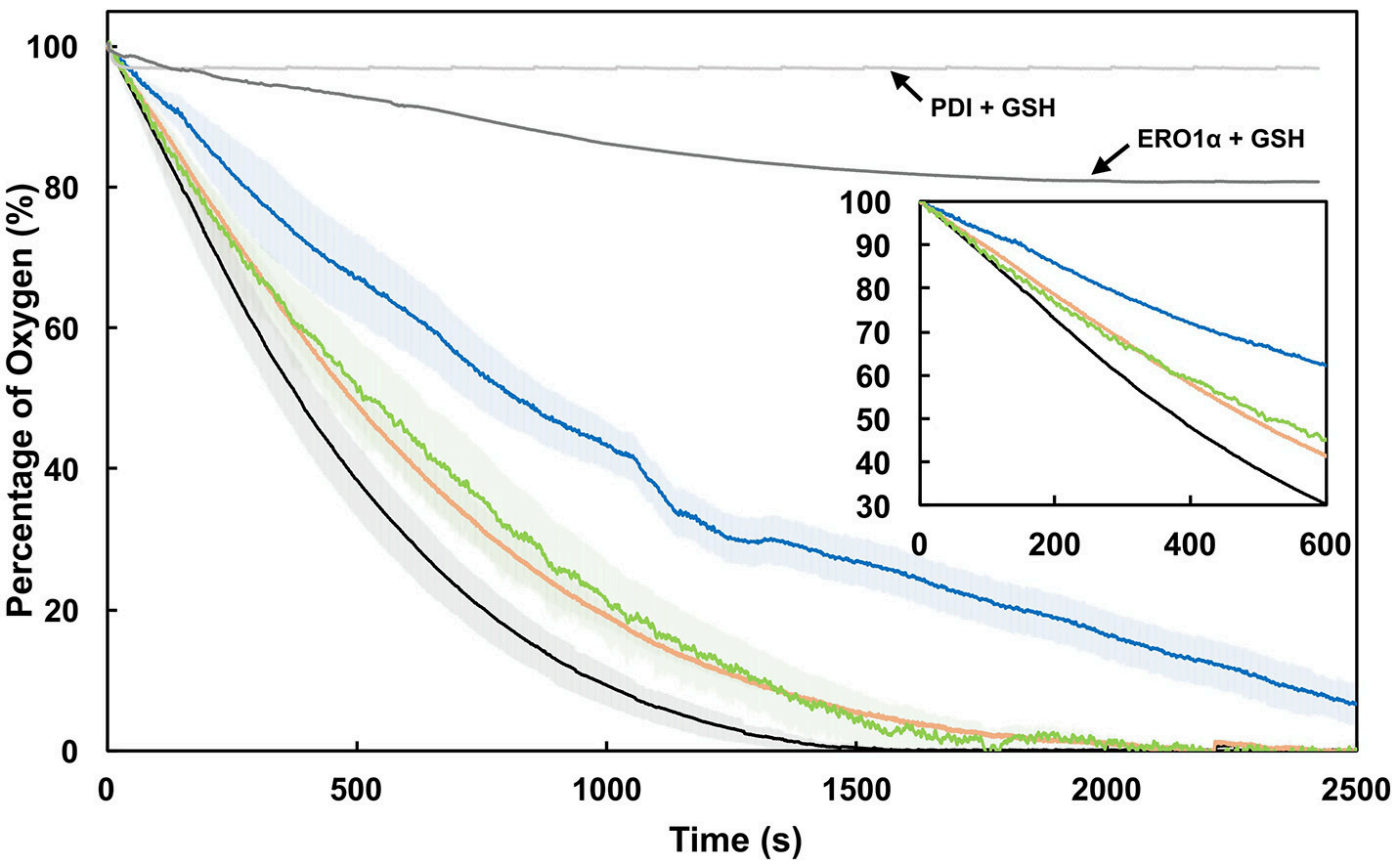
Mutation of an active site lysine residue impedes electron shuttling between PDI and ERO1α. Oxygen consumption by 2 μM ERO1α in the presence of 5 μM wild type PDI (*WT*; black), K57/401Q (blue), K57Q (green), or K401Q (orange) with 20 mM GSH. Shaded regions represent the standard deviation of the mean of three experiments. As controls, the rate of oxygen consumption was determined for 5 μM PDI in the presence of 20 mM GSH and for 2 μM ERO1α in the presence of 20 mM GSH. All experiments were performed in air-saturated ERO1α assay buffer at pH 7.4.

We hypothesized that the attenuation of di-E-GSSG reduction displayed by our PDI variants would effectively translate to the attenuation of electron shuttling between PDI and ERO1α; thus presenting a functional *in vivo* relevance to oxidative protein folding pathways and the intraluminal redox equilibria. To test this, the sulfhydryl oxidase activity of ERO1α was assessed by monitoring the catalytic consumption of oxygen in the presence of wild type PDI and the K-to-Q variants at pH 7.4. The ERO1α enzyme used was a constitutively active mutant lacking the regulatory cysteines Cys^104^ and Cys^131^ (C104/131A) (Chambers et al., 2010; Inaba et al., 2010; Araki and Nagata). Control rates of oxygen consumption were established for GSH+ERO1α in the absence of PDI (Figure 6, dark gray line) and for GSH+PDI in the absence of ERO1α (Figure 6, light gray line). In the presence of wild type PDI, ERO1α displayed the highest rate of oxygen consumption at ~0.12 ± 0.0057 s^−1^ (Figure 6, black line; Table 2). In contrast, in the presence of the K57/401Q double substitution variant (Figure 6, blue line) ERO1α was only capable of a rate of oxygen consumption that was ~2-fold (46%) less than that generated in the presence of wild type PDI. The single K-to-Q variants supported oxidase rates that were very similar, at 76 and 83% of wild type for K57Q and K401Q, respectively. Since electron transfer between PDI and ERO1α is step-wise (Araki et al., 2013), we can view these results mechanistically. The 24% decrease to the rate of oxygen consumption observed for ERO1α in the presence of the K57Q PDI variant is a result of the hindrance of the intramolecular thiol-disulfide exchange reaction that occurs between the reduced CGHCQ motif of the *a*-domain and the oxidized CGHCK motif of the *a*′-domain (Supplementary Figure 9, *step 2*). Furthermore, the observed 17% decrease of the rate when ERO1α is in the presence of the K401Q PDI variant was likely not a result of any impedance to the intramolecular thiol-disulfide exchange between the PDI *a*-type domains. Rather, reduction of the C^94^-C^99^ electron shuttling disulfide of ERO1α by PDI's *a*′-domain active site was likely the attenuated step (Supplementary Figure 9, *step 3*). In each case it is the rate of disulfide reduction that is attenuated. This interpretation makes sense when considering the altered active site conformation observed with a CGHCQ motif in comparison to CGHCK, as well as the implications of a less nucleophilic Cys_N_.

**Table 2:** Observed rates of ERO1α oxidase activity in the presence of wild type (*WT*) and the K-to-Q PDI variants.

**PDI**	**k_obs_ (s^−−1^)**	**%^a^**
WT	0.12 ± 0.0057	100
K57/401Q	0.064 ± 0.011^***^	54
K57Q	0.091 ± 0.011^*^	76
K401Q	0.10 ± 0.0035^*^	83

*Results were determined from the slopes of the initial linear phase of the oxygen consumption profiles in Figure 6. Results shown represent the mean ± SD of three experiments*.

a *Percentage difference of the mean relative to wild type*.

* P < 0.05,

*** *P < 0.005*.

As for the double substitution K57/401Q PDI variant, a combination of the mechanisms suggested for the single substitution variants led to the observed 2-fold decrease to the rates of oxygen consumption. Substitution of Lys^57^ and Lys^401^ resulted in markedly slowed rates of electron transfer to ERO1α. These active site residues are therefore viewed as highly important for the ERO1α-mediated regeneration of oxPDI, whereby absence of the native CGHCK motif effectively forms a kinetic bottleneck, limiting the efficiency of electron transfer. Physiologically, this implies a possible build-up of unfolded proteins leading to reductive ER stress; a particularly damaging result in professional secretory cells (Back and Kaufman, 2012; Hudson et al., 2015). Not only does a kinetically abated PDI enzyme affect the reaction with ERO1α, but this result may indicate that it is more than likely to negatively impact other ER-luminal enzymes important to protein folding, such as peroxiredoxin and quiescin sulfhydryl oxidase. On the subcellular level PDI is nearly ubiquitous and attenuation of its activity as centered on modifications (or substitutions) of Lys^57^ and Lys^401^ offers seemingly endless possibilities to perturbing the status of many intricate pathways.

### Lysine acetylation of PDI as a post-translational modulator of activity

To date, no naturally occurring mutations or disease-related single-nucleotide polymorphisms have been identified for human PDI at residues Lys^57^ and Lys^401^. Therefore, the PDI variants designed here do not accurately represent a physiologically relevant protein form. Thus, it seemed logical to consider Lys^57^ and Lys^401^ as regulatory centers and candidates for reversible acetylation (*N*^ε^-acetylation). This seemed particularly pertinent considering the recent advances in ER-acetylome work (refer to introduction) and the identification of acetylated Lys^401^ (Pehar et al., 2012). Contrarily, a recent acetylome analysis identified six lysine residues of PDI as targets for reversible acetylation; Lys^57^ and Lys^401^ were not found to be modified (Schölz et al., 2015). The discrepancy of these results is an indicator of a need for specific studies that focus on PDI, rather than omics, in an attempt to avoid sample complexity that could otherwise convolute the identification of modified residues, such as Lys^57^ and Lys^401^.

**Figure 7:**
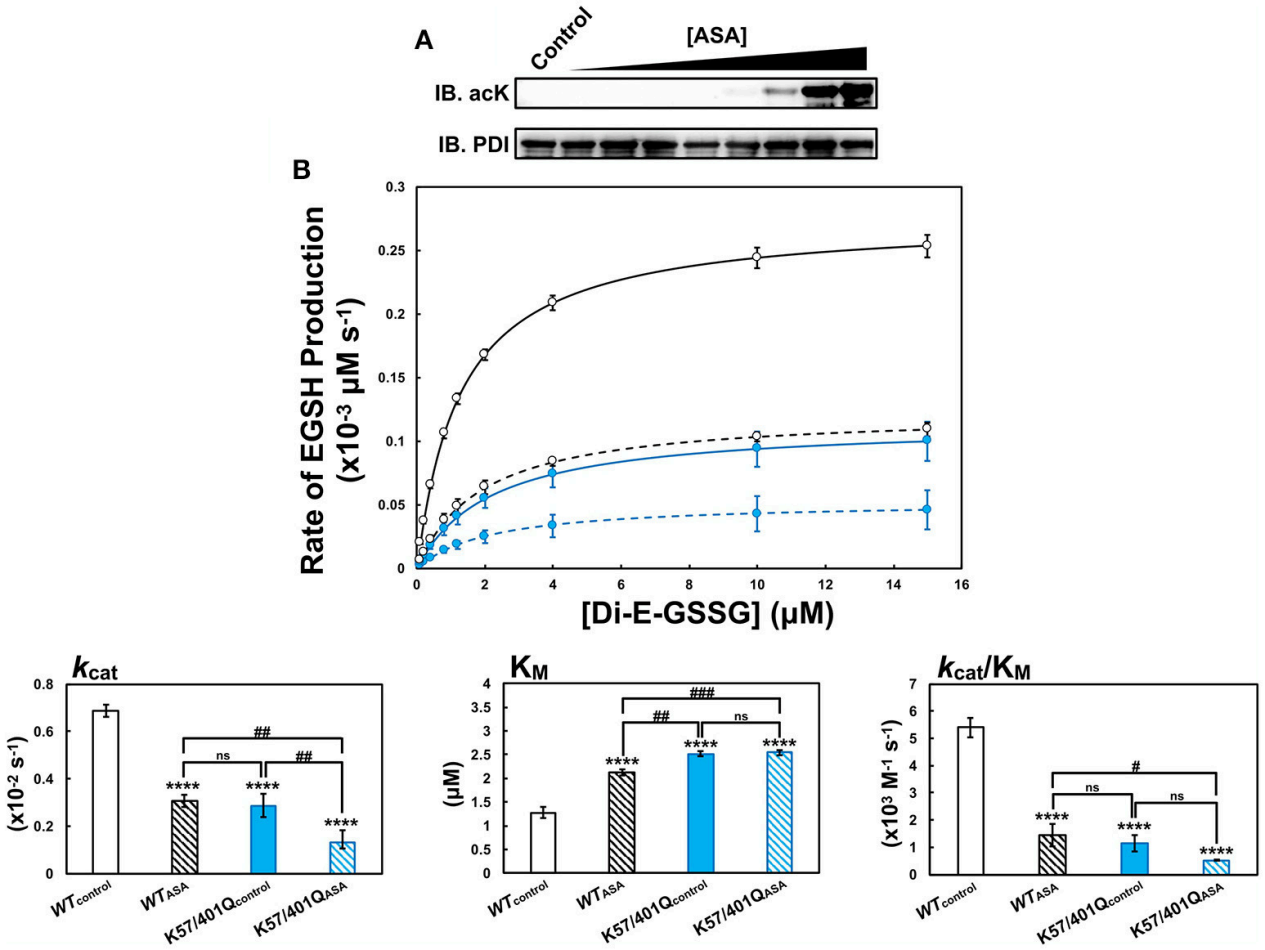
PDI is acetylated by aspirin (acetylsalicylic acid, ASA) *in vitro*. **(A)** Purified wild type PDI (*WT*, 2 μM) was treated with varying concentrations of ASA (0, 0.25, 0.5, 0.75, 1, 2, 5, 10, and 15 mM) for 4 h at 37°C in a 0.1 M Tris-HCl buffer pH 8.5. Immunoblotting was performed using an anti-acetyllysine pan-specific antibody for the detection of acetylated lysine residues and the anti-PDI RL90 antibody was used to determine consistent loading of PDI (500 ng). **(B)** PDI at a concentration of 2 μM was treated with 15 mM ASA for 4 h at 37°C in the presence of 0.5 mM DTT. Untreated, parallel controls were also performed in the absence of ASA. Using the di-E-GSSG assay, the steady-state oxidoreductase kinetics of 20 nM untreated control wild type (black solid line, *WT*_control_), ASA-treated wild type (black dotted line, *WT*_ASA_), untreated control K57/401Q (blue solid line, K57/401Q_control_), and ASA-treated K57/401Q (blue dotted line, K57/401Q_ASA_) were assayed at pH 7.4 in PDI assay buffer. Reported data represent the mean ± *SD* of three experiments. Asterisks (^*^) represent statistically significant differences between untreated wild type and the respective comparators. Hash-tags (^#^) denote statically significant differences between the indicated pairs.

To establish if the active site residues could be acetylated, an *in vitro* acetylation procedure was performed using acetylsalicylic acid (ASA), the active ingredient of the popular therapeutic aspirin. ASA acetylation of PDI was found to be dose-dependent, with as little as 2 mM ASA providing detectable levels of the modification as determined by western blot (Figure 7A).

Next, PDI was acetylated using 15 mM ASA by the same *in vitro* technique described, followed by mass spectrometry analysis to identify all acetyllysine (acLys) residues. Of the 48 lysine residues present in the PDI construct used (Supplementary Figure 1B), 28 acetyllysine residues (acLys) were identified. This included acetylated active site residues Lys^57^ and Lys^401^ (Supplementary Figure 11; Supplementary Spreadsheet). The presence of acLys^57^ and acLys^401^ was confirmed by tandem MS following enrichment of the thiol-containing active site peptides using gold nanoparticles (Supplementary Figure 10).

This *in vitro* acetylation approach resulted in a highly acetylated form of PDI. The catalytic *a* and *a*′ domains harbored nine and six acLys residues, respectively. The *b* and *b*′-domains each had five acLys residues. Additionally, the *x*-linker contained three acLys residues. Interestingly, alongside the active site lysine residues, Lys^81^ and Lys^424^ were also found to be acetylated by ASA. These have been implicated to form conserved Lys-Glu salt bridges peripheral to each respective active site to enable proton transfer during catalysis (Ellgaard and Ruddock, 2005) (Supplementary Figure 11).

It should be noted that the acetylation reaction employed likely resulted in a relatively low stoichiometry of acLys. First, in aqueous conditions ASA is susceptible to hydrolysis resulting in salicylic acid and acetate. Secondly, ASA is not purely amine-directing. It is possible to observe *O*-acetylation as well (Botting, 2010). For the purposes of this study, *O*-acetylation of PDI was reverted by incubation with HA. Nevertheless, these possibilities would effectively limit the stoichiometry of lysine acetylation via direct competition and sequestration of ASA. This was confirmed by the presence of non-acetylated active site peptides (for the *a*-domain *m/z* 846.8987, *z* = 2; and for the *a*′-domain *m/z* 668.9878, *z* = 3, results not shown).

The effect of ASA-mediated acetylation on the steady-state oxidoreductase kinetics of PDI was assessed using the di-E-GSSG assay (Figure 7B). To discern the impact of acetylating Lys^57^ and Lys^401^ in relation to the other acLys residues generated from the *in vitro* treatment with ASA, activity of ASA-acetylated wild type PDI (*WT*_ASA_, Figure 7B, black dotted line) was compared to ASA-acetylated K57/401Q (K57/401Q_ASA_, Figure 7B, blue dotted line). Negative controls consisted of non-acetylated wild type (*WT*_control_, Figure 7B, black solid line) and non-acetylated K57/401Q (K57/401Q_control_, Figure 7B, blue solid line). Due to the *in vitro* reaction yielding imperfect acLys stoichiometry, a heterogeneous mixture of acPDI molecules can be expected (*i.e*., no acetylation of Lys^57^ and Lys^401^, partial acetylation of either Lys^57^ or Lys^401^ and complete acetylation of each active site lysine per PDI molecule). Thus, the results should be taken with consideration for the presence of non-acetylated active site lysine residues that persist to some extent for ASA-acetylated wild type.

As illustrated by Figure 7B (summarized in Supplementary Table 7), non-acetylated wild type was the superior oxidoreductase. ASA-acetylated wild type, non-acetylated K57/401Q and ASA-acetylated K57/401Q all exhibited very similar kinetic properties. The enzymatic activity of ASA-acetylated wild type and non-acetylated K57/401Q displayed stark similarity with a substantial decrease of substrate affinity (K_M_) and turn over (*k*_cat_) by ~2-fold in comparison to non-acetylated wild type. Taken together, the overall efficiency of PDI as an oxidoreductase when treated with aspirin was diminished 4-fold. This corroborated closely with those previous results observed in Figure 3. Based on this, the effects of lysine acetylation likely closely mimic the effects of substitution by glutamine. That being the change of the active site conformation as observed in Figure 4. Acetylating the K57/401Q double substitution variant (K57/401Q_ASA_) provided insight as to the kinetic perturbations imposed by acetylation at lysine residues other than those flanking the active site. In particular, the primary effect was on *k*_cat_. This indicates that the acetylation state of PDI following aspirin treatment, coupled with the complete modification to Lys^57^ and Lys^401^ (substitution to Gln) generates an enzyme greater than 10-fold less efficient in comparison to non-acetylated wild type. As mentioned, of the acetylated lysine residues identified, acLys^81^ and acLys^424^ are likely candidates to lend some adverse kinetic effects. Although, it remains that acetylation of Lys^57^ and Lys^401^ by-and-large dictates the attenuation of oxidoreductase activity. Due to the heterogeneous acetylation produced using this *in vitro* procedure, we are placing a focus on genetically encoding site-specific acetyllysine residues at positions 57 and 401 for future studies (Neumann et al., 2009; Wang et al., 2017).

## Conclusions

In this study, we have used a combination of site specific mutagenesis and comprehensive kinetic analyses to demonstrate the importance of the PDI active site-flanking Lys^57^ and Lys^401^ to both oxidoreductase and thiol-reductase activity. We postulate that these highly conserved residues optimize the active site conformation by providing seemingly enhanced mobility to the loop region. This motion is believed to impose on the CxxC-intervening histidine thereby preventing steric hindrance of the active site cysteines, in particular, the N-terminal nucleophilic cysteine. The post-translational modification of lysine acetylation is a fruitful candidate for the regulation of PDI activity as it likely limits the proposed active site motion, thus resulting in attenuated oxidoreductase and thiol-reductase activity. This study provides a strong indication that acetylation of the active site-flanking lysine residues can act to reversibly regulate the activity of PDI. Our results justify further investigation of the potential to modulating PDI activity by reversible acetylation of the active site lysine residues with a focus on the physiological implications of such.

## Author contributions

CC, HA, and BM: Designed the research; CC, HA, and JA: Performed the experiments; CC and BM: Analyzed the data; RU, KA, and KN: Provided the recombinant ERO1α construct; CC and BM: Wrote the paper.

### Conflict of interest statement

The authors declare that the research was conducted in the absence of any commercial or financial relationships that could be construed as a potential conflict of interest.

## Abbreviations

PDI: protein disulfide isomerase
di-E-GSSG: dieosin glutathione disulfide
acPDI: acetylated PDI
acLys: acetyllysine
ASA: acetylsalicylic acid
ER: endoplasmic reticulum
ERO: endoplasmic reticulum oxidoreductin
WT: wild type
FSQ: fluorescence self-quenching
FAD: flavin adenine dinucleotide
DTT: dithiothreitol.
