# Supplementary material for: Conserved Residues Lys57 and Lys401 of Protein Disulfide Isomerase Maintain an Active Site Conformation for Optimal Activity: Implications for Post-Translational Regulation
Source: Front Mol Biosci. 2018 Feb 28;5:18. doi: 10.3389/fmolb.2018.00018 (PMC5835755; doi:10.3389/fmolb.2018.00018)
Supplement: Supplementary file 1 [file DataSheet1.DOCX]

Supplementary Material

**Conserved Residues Lys^57^ and Lys^401^ of Protein Disulfide Isomerase Maintain an Active Site Conformation for Optimal Activity: Implications for Post-Translational Regulation**

**Cody Caba, Hyder Ali Khan, Janeen Auld, Ryo Ushioda, Kazutaka Araki, Kazuhiro Nagata, Bulent Mutus^*^**

*** Correspondence:** Bulent Mutus: [mutusb@uwindsor.ca](mailto:mutusb@uwindsor.ca)

**Method for Supplementary Figure 5C**

To trap PDI in a mixed disulfide state with the fluorescent eosin-glutathione substrate (EGSH), iodoacetamide (IAM)-mediated thiol blocking was utilized. Under similar steady-state conditions as employed previously, 150 nM PDI was incubated with 5 μM di-E-GSSG in PDI assay buffer of varying pH containing 10 μM DTT. The reaction was quenched at defined time points by the addition of 30 mM IAM for a 20 min incubation at room temperature. PDI was separated from unreacted di-E-GSSG, free EGSH and excess IAM by buffer exchange to PDI assay buffer (pH 7.4) using Zeba Spin Desalting columns (MWCO: 7 kDa) as per the manufacturer's instructions. Samples were then loaded to a black, opaque fluorescence-optimized 96-well plate (Corning^TM^) for fluorescent measurements using a SpectraMax M5E microplate reader (λ_ex_ 525 nm; λ_em_ 545 nm). The fluorescence readings were related to an EGSH standard curve to quantify the amount of PDI active sites trapped in a mixed-disulfide intermediate state based on the fluorescence carry-over following buffer exchange.

## Methods for sample preparation and acquisition of mass spectrometry data (Supplementary spreadsheet; Supplementary Figure 10,11)

Digestion of affinity purified PDI followed an in-solution protocol using Glu-C endopeptidase (Promega). 100 μg of PDI was diluted 1:1 with 50 mM ammonium bicarbonate buffer pH 8.0 (ABC, pH unadjusted) containing 4 M urea. To this, 2 mM DTT was added and allowed to fully reduce PDI disulfides for 30 min at 60 ^O^C. Fully reduced PDI was desalted and buffer exchanged to 25 mM ABC pH 8.0 using Zeba Spin Desalting columns (MWCO: 7 kDa). Glu-C was added to 30 μg of PDI at a protein:protease ratio of 10:1 and incubated for 16 h at room temperature with shaking. Proteolysis was quenched by the addition of 1% formic acid (FA) and the peptides were desalted using C_18_ ZipTips (EMD Millipore) according to the manufacturer. Peptides were dried and resuspended in 0.1% FA for ESI-MS analysis. An unquenched fraction of the digest was saved for the purpose of enrichment of thiol-containing peptides. Enrichment of thiol-containing peptides using gold nanoparticles (AuNPs) was performed as described previously (Freeman et al., 1995, Faccenda et al., 2010). Samples were analyzed using a Waters SYNAPT G2-Si time-of-flight mass spectrometer configured for nano-ESI operated in positive-ion mode coupled to a Waters nanoACQUITY UPLC system (UPLC-ESI MS). The UPLC was configured for 1D single pump trapping with a Waters ACQUITY UPLC Symmetry C_18_ 2G V/M trap column coupled with a Waters ACQUITY Peptide BEH C_18_ with 0.5 to 1 μl sample injections. Mobile phase buffer A was 0.1% FA. Mobile phase buffer B was acetonitrile with 0.1% FA. Samples were loaded on the trap column for 3 min at a flow rate of 5 μl/ min in 97% buffer A, 3% buffer B. A 7-step, 60 min gradient was run at a flow rate of 0.3 μl/ min where buffer A concentration was 97% at 0 min to 90% at 5 min, 75% at 33 min, 50% at 41 min, 15% at 42 min to 44 min, and 97% at 45 min to 60 min. Samples were first analyzed in MS^e^ mode prior to MS/MS sequencing. MS/MS followed a data-dependent acquisition method by targeting the precursor ions of interest identified by MS^e^.

Peptide identification from MS^e^ data was performed using the Waters' ProteinLynx Global SERVER. The processing parameters used were as follows: low energy threshold of 135 counts, high energy threshold of 30 counts and post-acquisition lock mass correction with a tolerance of 0.25. Database searches were performed against the known sequence of wild type (*WT*) PDI (supplementary Figure 1B) and the UniProtKB source database for *Escherichia coli* (in consideration of recombinant PDI being overexpressed and isolated from *E. coli*)*.* The parameters were as follows: a minimum of six amino acids per peptide fragment, minimum three fragments per peptide, maximum protein mass of 250 kDa, maximum two missed cleavages (Glu-C), variable modifications of methionine oxidation and lysine acetylation were specified.

**Supplementary Table A.1**: PCR primers for site-directed mutagenesis of hPDI. Substituted nucleotides are in bold and underlined. T_a_ represents the primer annealing temperature used.

| **Primer** | **Sequence (5' – 3')** | **T_a_ (^O^C)** |
| --- | --- | --- |
| a-domain rev. | AGG GGC CAG AGC CGC GCA GTG GCC ACA CCA AGG | 61-68 |
| K57A a-domain fwd. | AGG GGC CAG AGC C**GC** GCA GTG GCC ACA CCA AGG | 65 |
| K57Q a-domain fwd. | AGG GGC CAG AGC CT**G** GCA GTG GCC ACA CCA AGG | 68 |
| K57E a-domain fwd. | AGG GGC CAG AGC CT**C** GCA GTG GC | 61 |
| a'-domain rev. | CCA TGG GGC ATA GAA TTC CAC AAA GAC | 57-60 |
| K401A a'-domain fwd. | TGT GGT CAC TGC **GC**A CAG TTG GCT CCC | 57 |
| K401Q a'-domain fwd. | TGT GGT CAC TGC **C**AA CAG TTG GCT CCC | 60 |
| K401E a'-domain fwd. | TGT GGT CAC TGC **G**AA CAG TTG GCT C | 58 |
| CxxCK^57^–AxxAK^57^ fwd. | AGG GGC CAG AGC CTT G**GC** GTG GCC A**GC** CCA AGG GGC ATA G | 58 |
| CxxCK^57^–AxxAK^57^ rev. | AGG GGC CAG AGC CGC GCA GTG GCC ACA CCA AGG | 61-68 |
| CxxCK^401^–AxxAK^401^ fwd. | CTA TGC CCC ATG G**GC** TGG TCA C**GC** CAA ACA GTT GGC TCC | 54 |
| CxxCK^401^–AxxAK^401^ rev. | AAC TCC ACA AAG ACG TTT TTT TTC TCA TC | 54 |


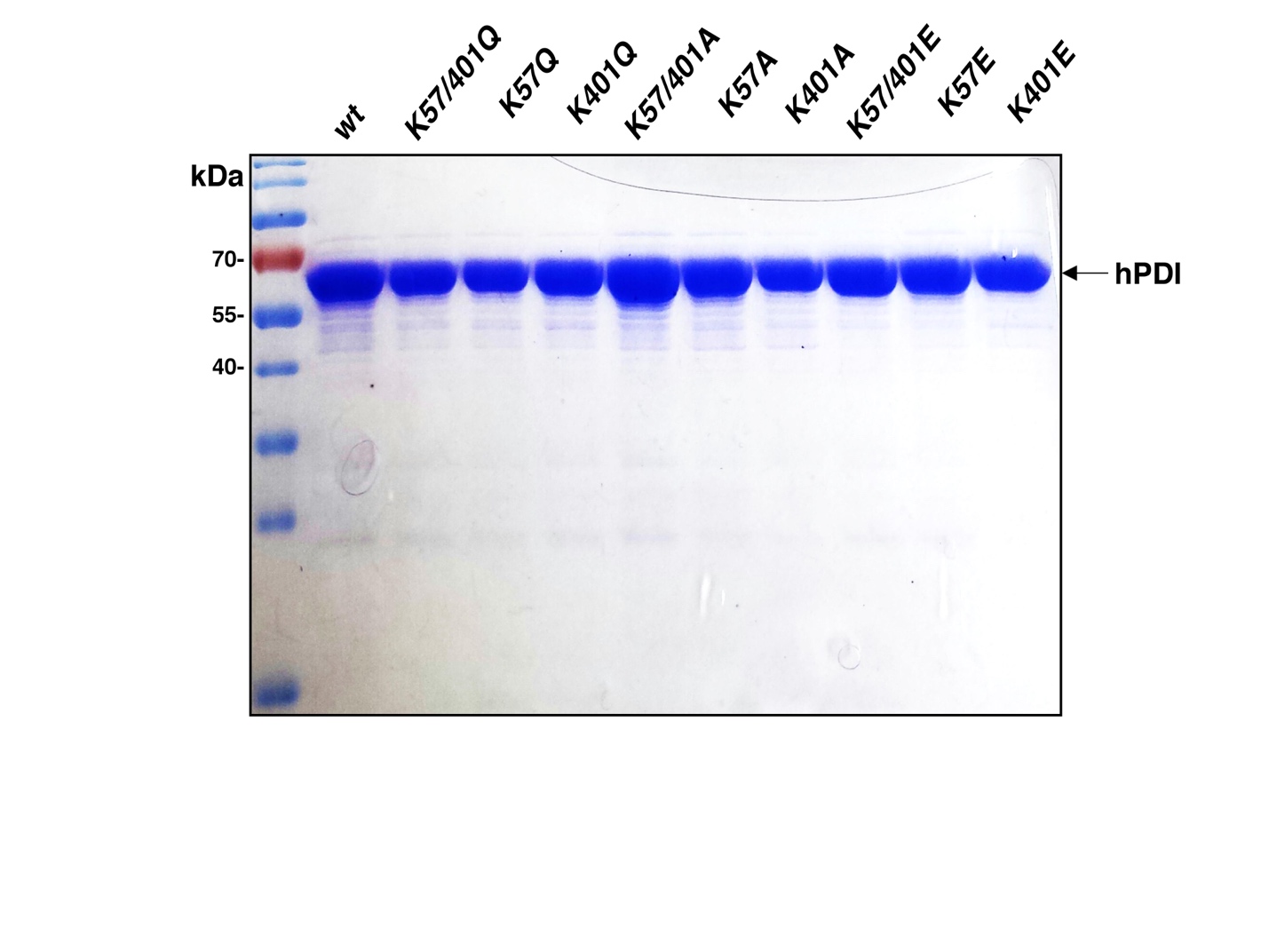


**B**

**A**

MGSSHHHHHH SSGLVPRGSH MASMTGGQQM GRDPNSDAPE EEDHVLVLRK SNFAEALAAH KYLLVEFYAP W**CGHCK^57^**ALAP EYAKAAGKLK AEGSEIRLAK VDATEESDLA QQYGVRGYPT IKFFRNGDTA SPKEYTAGRE ADDIVNWLKK RTGPAATTLP DGAAAESLVE SSEVAVIGFF KDVESDSAKQ FLQAAEAIDD IPFGITSNSD VFSKYQLDKD GVVLFKKFDE GRNNFEGEVT KENLLDFIKH NQLPLVIEFT EQTAPKIFGG EIKTHILLFL PKSVSDYDGK LSNFKTAAES FKGKILFIFI DSDHTDNQRI LEFFGLKKEE CPAVRLITLE EEMTKYKPES EELTAERITE FCHRFLEGKI KPHLMSQELP EDWDKQPVKV LVGKNFEDVA FDEKKNVFVE FYAPW**CGHCK^401^** QLAPIWDKLG ETYKDHENIV IAKMDSTANE VEAVKVHSFP TLKFFPASAD RTVIDYNGER TLDGFKKFLE SGGQDGAGDD DDLEDLEEAE EPDMEEDDDQ KAVKDELKLA AALEHHHHHH

**Supplementary Figure 1. (A)** Purity of isolated His_6_-PDI; wild-type (*WT*) and mutant variants was assessed via SDS-PAGE using a 10% polyacrylamide gel and visualized by Coomassie staining. N- and C-terminally His_6_-tagged PDI (~59 kDa) is indicated with reference to a standard molecular weight ladder. **(B)** Amino acid sequence of the recombinant, mature, human PDI used in this study. Active sites (CGHCK) of the *a*- and *a'*-domains are underlined and in bold.


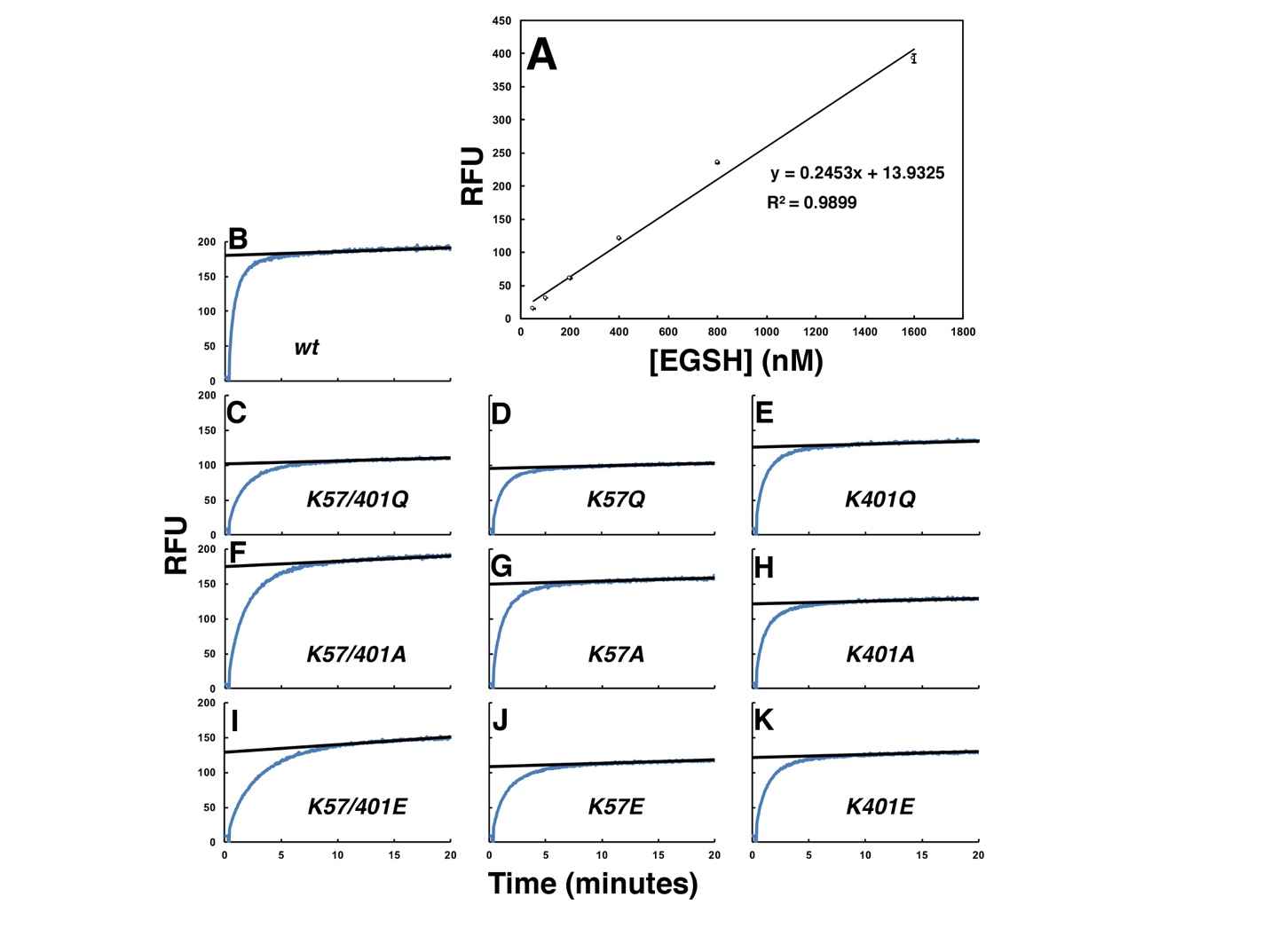


**Supplementary Figure 2.** Determining the concentration of catalytically active PDI by burst-phase analysis. In a cuvette, 10 ul of purified enzyme (16.9 μM; 1 mg/ml stock) was incubated with 800 nM di-E-GSSG in PDI assay buffer (final volume 500 ul). **(B-K)** The increase of fluorescence (relative fluorescence units; RFU) was monitored over 20 min for each PDI variant indicated. Following the initial burst, linear regression analysis provided quantitation of the fluorescence increase (y-intercept) that was corrected with the base-line fluorescence of the self-quenching di-E-GSSG molecules. The corrected y-intercept of each respective plot from **B-K** was used to determine the concentration of functional enzyme by relating the enzyme-mediated fluorescence output to the concentration of EGSH produced using a standard curve. **(A)** The standard curve of fluorescence *vs.* the concentration of EGSH was generated by complete reduction of di-E-GSSG using 1 M DTT in PDI assay buffer. Burst-phase data are representative of an experiment performed in triplicate. The standard curve represents the mean ± S.D. of three technical replicates.

*
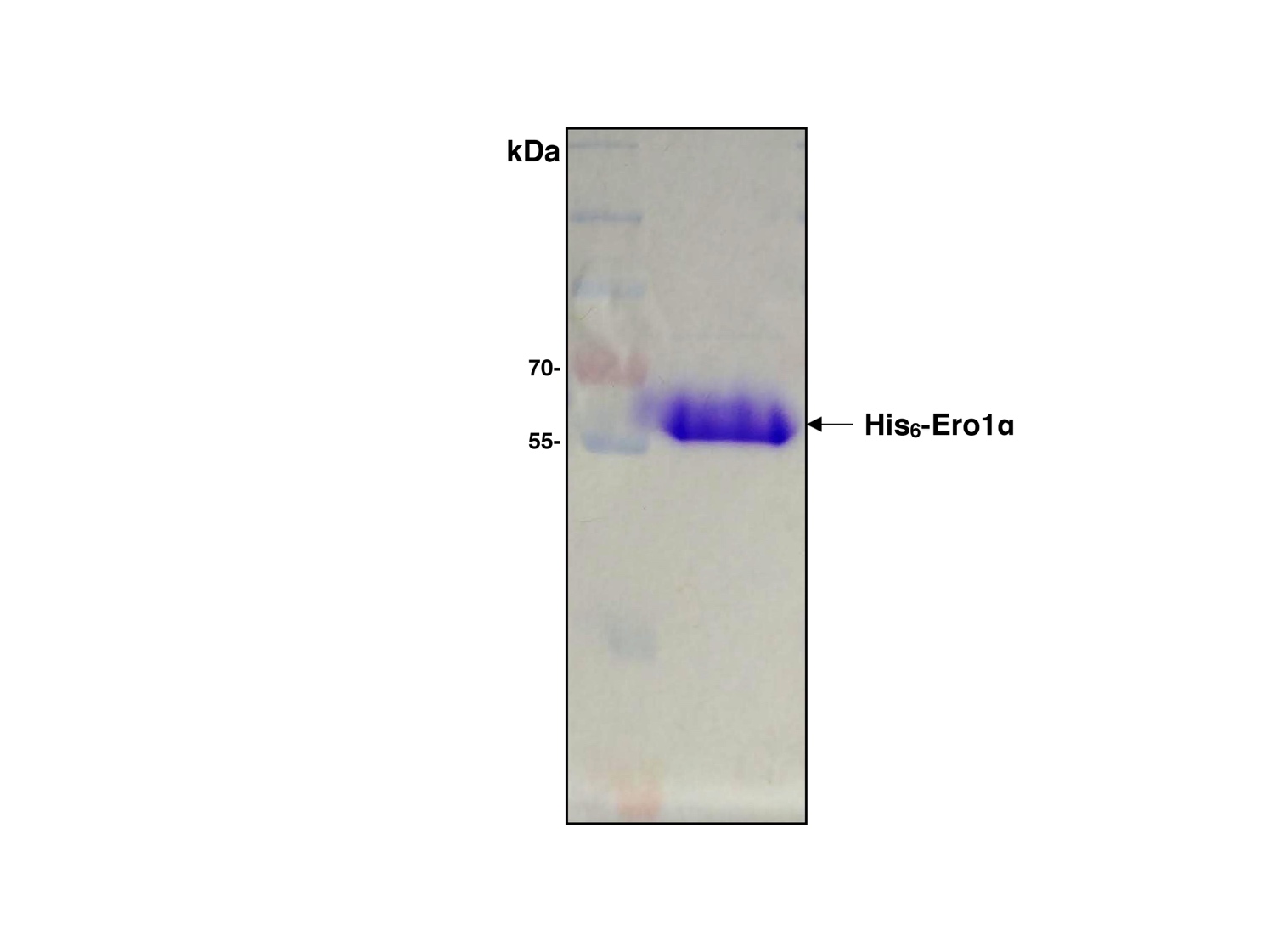
*

**Supplementary Figure 3.** Purity of affinity purified endoplasmic oxidoreductin 1α (ERO1α) was assessed via SDS-PAGE using a 10% polyacrylamide gel and visualized by Coomassie staining. N-terminally 6-His tagged ERO1α (~55 kDa) is indicated with reference to a standard molecular weight ladder.

**B**

**A**


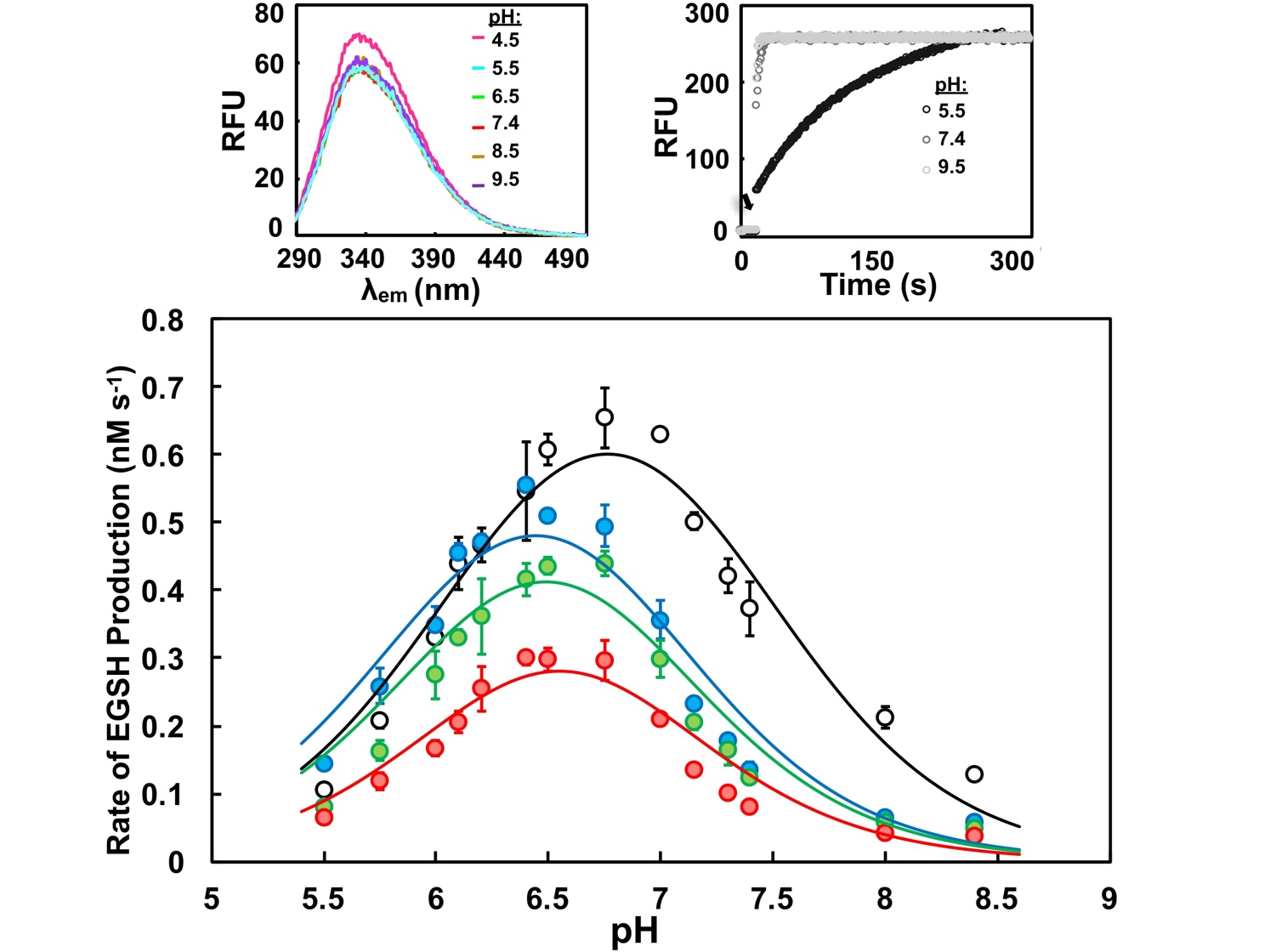


**Supplementary Figure 4. (A)** Tryptophan fluorescence (RFU) of 6.175 μM redPDI was monitored over the indicated pH range as a reporter of pH-induced conformational changes. **(B)** The di-E-GSSG probe maintained its fluorescence properties over the tested pH range. The fluorescence fold increase of 800 nM di-E-GSSG was monitored after the addition of 1 M DTT (black arrow). The retarded rate of reduction at pH 5.5 (black circles) is attributed to DTT being only ~0.02% deprotonated (more reactive thiolate form) as a result of its thiol p*K*_a_'s (*ca.* 9.2).


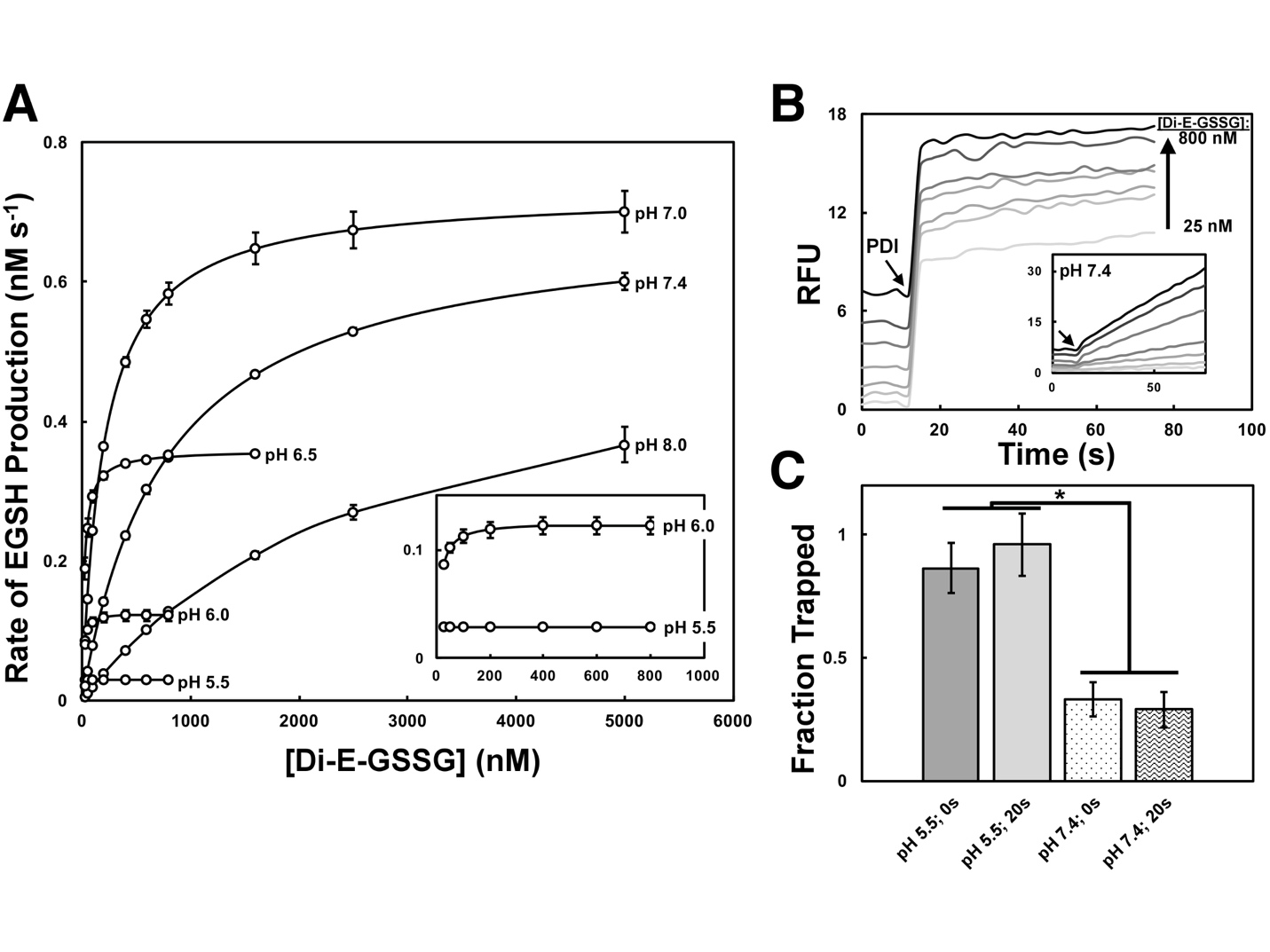


**Supplementary Figure 5.** PDI undergoes substrate trapping via markedly attenuated substrate turnover at acidic pH. **(A)** Representative plots of the Michaelis-Menten kinetic relationships over the range of pH tested for the reduction of di-E-GSSG by 10 nM PDI in the presence of 10 μM DTT. **(B)** Raw data of the fluorescence (RFU) increase over time depicting a comparison of the initial rates of catalysis at pH 5.5 and 7.4 (*inset*). The addition of wild type PDI (10 nM) to the reaction mixture is indicated by an arrow. **(C)** The impact of pH on PDI-substrate mixed-disulfide complex trapping was assessed under steady-state conditions similar to those of the kinetic assays of **A**, with the addition of iodoacetamide (IAM) as the "trapping" reagent. Results are reported as the fraction of trapped PDI active sites as determined by the fluorescence carry-over after protein desalting and reference to a standard curve of fluorescent signal *vs.* [EGSH]. Data from **A** and **C** represent the mean ± S.D. of three experiments.


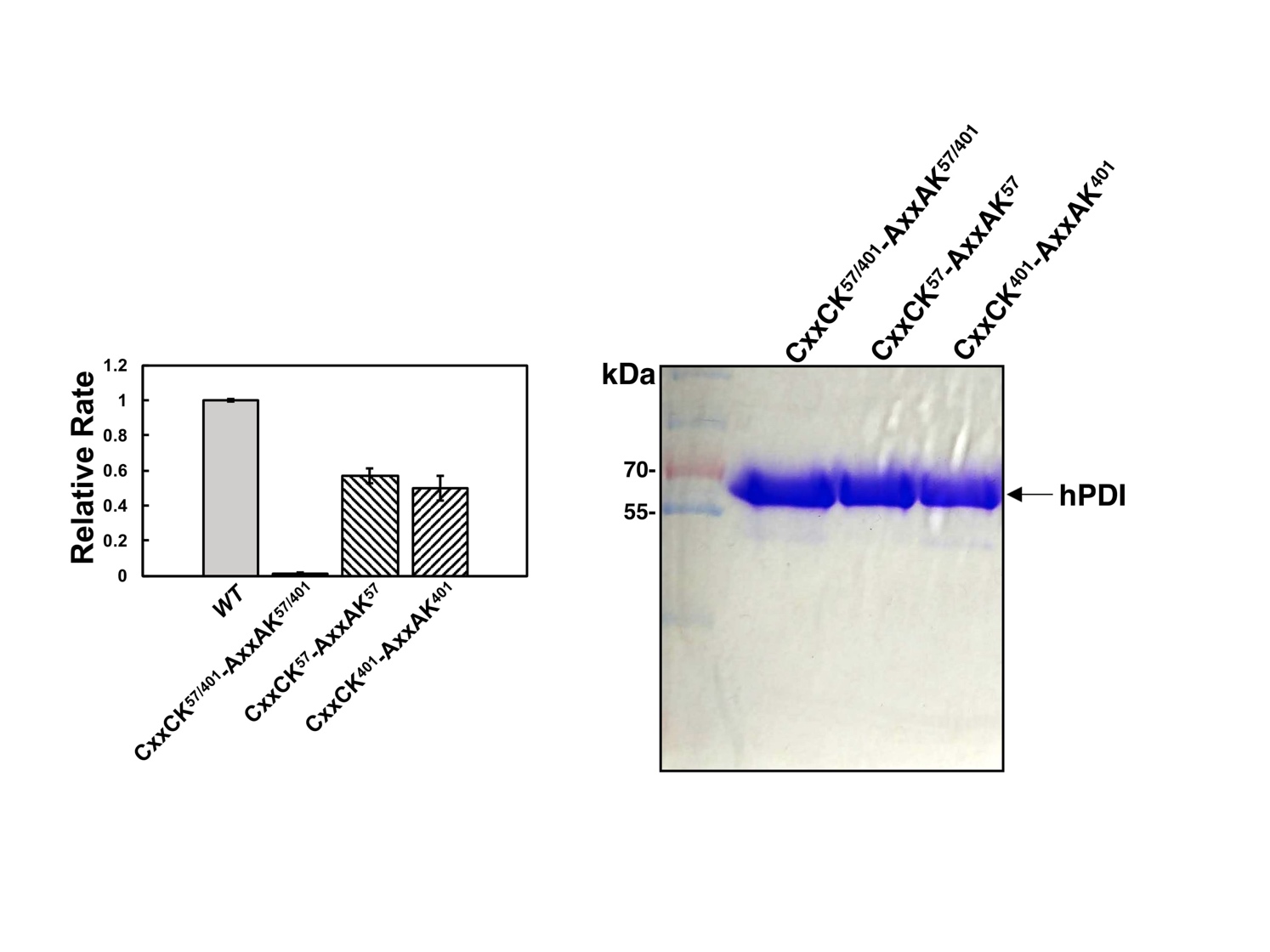


**B**

**A**

**Supplementary Figure 6. (A)** Using the di-E-GSSG (500 nM) assay the activity of 10 nM of each mutant was assayed in relation to wild type (*WT*) as a simple reporter of active site inactivation due to cysteine-to-alanine substitution of the vicinal thiols of the respective motif(s). Substitution of a single active site cysteine pair resulted in a near-loss of 50% activity, while mutation of both active sites rendered PDI inactive (CxxCK^57/401^-AxxAK^57/401^).  **(B)** Purity of affinity purified hPDI active site thiol mutants (CxxC-AxxA) was assessed via SDS-PAGE using a 10% polyacrylamide gel and visualized by Coomassie staining. N- and C-terminally 6-His tagged hPDI (~59 kDa) is indicated with reference to a standard molecular weight ladder.

**Supplementary Table 2.** Apparent steady-state kinetic parameters at pH 6.0. Results are representative of the mean ± S.D. of three experiments. Data are also presented as the percentage difference of the mean compared to wild type (*WT*).

| **PDI** | ***k*_cat_ (×10^-2^ s^-1^, %^a^)** | **K_M_ (μM, %^a^)** | ***k*_cat_/K_M_ (×10^3^ M^-1^ s^-1^, %^a^)** |
| --- | --- | --- | --- |
| *WT* | 0.627 ± 0.043, 100 | 0.0107 ± 0.00150, 100 | 588 ± 31.5, 100 |
| K57/401Q | 0.720 ± 0.028, 115 | 0.0203 ± 0.00265, 190 **^*^** | 355 ± 63.4, 60 |
| K57Q | 0.719 ± 0.056, 115 | 0.0136 ± 0.00334, 128 | 529 ± 118, 90 |
| K401Q | 0.695 ± 0.010, 110 | 0.0103 ± 0.00225, 97 | 675 ± 220, 115 |
| K57/401A | 0.610 ± 0.099, 92 | 0.0113 ± 0.00331, 106 | 539 ± 57.0, 92 |
| K57A | 0.640 ± 0.050, 84 | 0.0130 ± 0.00663, 122 | 492 ± 110, 84 |
| K401A | 0.695 ± 0.021, 117 | 0.0101 ± 0.00540, 95 | 687 ± 220, 117 |
| K57/401E | 0.434 ± 0.012, 76 **^***^** | 0.0965 ± 0.00095, 94 | 449 ± 65.4, 76 |
| K57E | 0.598 ± 0.041, 89 | 0.0114 ± 0.00223, 107 | 525 ± 86.4, 89 |
| K401E | 0.715 ± 0.048, 92 | 0.0132 ± 0.00294, 124 | 540 ± 97.2, 92 |

**^a^** Relative to wild type

**Supplementary Table 3.** Apparent steady-state kinetic parameters at pH 6.5. Results are representative of the mean ± S.D. of three experiments. Data are also presented as the percentage difference of the mean compared to wild type (*WT*).

| **PDI** | ***k*_cat_ (×10^-2^ s^-1^, %^a^)** | **K_M_ (μM, %^a^)** | ***k*_cat_/K_M_ (×10^3^ M^-1^ s^-1^, %^a^)** |
| --- | --- | --- | --- |
| *WT* | 1.79 ± 0.0133, 100 | 0.0203 ± 0.00279, 100 | 882 ± 90.9, 100 |
| K57/401Q | 2.30 ± 0.0807, 128 **^****^** | 0.0678 ± 0.01047, 334 **^****^** | 340 ± 57.0, 39 **^****^** |
| K57Q | 2.56 ± 0.0848, 143 **^****^** | 0.0477 ± 0.00695, 235 **^***^** | 537 ± 78.3, 61 **^***^** |
| K401Q | 2.30 ± 0.140, 128 **^****^** | 0.0428 ± 0.00701, 211 **^***^** | 537 ± 55.7, 61 **^***^** |
| K57/401A | 1.77 ± 0.0611, 99 | 0.0427 ± 0.00863, 210 **^***^** | 414 ± 75.9, 47 **^****^** |
| K57A | 1.68 ± 0.0001, 94 | 0.0290 ± 0.00025, 143 | 579 ± 44.0, 66 **^**^** |
| K401A | 1.89 ± 0.0477, 105 | 0.0318 ± 0.00396, 157 | 590 ± 79.0, 67 **^***^** |
| K57/401E | 1.42 ± 0.0880, 79 **^**^** | 0.0311 ± 0.00414, 153 | 455 ± 83.5, 52 **^****^** |
| K57E | 1.89 ± 0.233, 105 | 0.0308 ± 0.00294, 152 | 612 ± 70.2, 69 **^**^** |
| K401E | 2.04 ± 0.0672, 113 **^*^** | 0.0357 ± 0.00239, 176 **^*^** | 571 ± 83.2, 65 **^***^** |

**^a^** Relative to wild type

**Supplementary Table 4.** Apparent steady-state kinetic parameters at pH 7.0. Results are representative of the mean ± S.D. of three experiments. Data are also presented as the percentage difference of the mean compared to wild type (*WT*).

| **PDI** | ***k*_cat_ (×10^-2^ s^-1^, %^a^)** | **K_M_ (μM, %^a^)** | ***k*_cat_/K_M_ (×10^3^ M^-1^ s^-1^, %^a^)** |
| --- | --- | --- | --- |
| *WT* | 3.64 ± 0.164, 100 | 0.191 ± 0.0178, 100 | 190 ± 8.05, 100 |
| K57/401Q | 3.08 ± 0.0479, 85 **^****^** | 0.522 ± 0.0134, 273 **^****^** | 59.0 ± 5.61, 31 **^****^** |
| K57Q | 3.97 ± 0.123, 109 **^*^** | 0.347 ± 0.0235, 181 **^****^** | 114 ± 5.08, 60 **^****^** |
| K401Q | 4.21 ± 0.0338, 116 **^****^** | 0.374 ± 0.0227, 195 **^****^** | 113 ± 7.40, 59 **^****^** |
| K57/401A | 2.48 ± 0.0522, 68 **^****^** | 0.397 ± 0.0131, 207 **^****^** | 62.4 ± 2.48, 33 **^****^** |
| K57A | 3.31 ± 0.0313, 91 **^*^** | 0.288 ± 0.0106, 150 **^***^** | 115 ± 3.50, 60 **^****^** |
| K401A | 3.46 ± 0.193, 95 | 0.306 ± 0.0468, 160 **^****^** | 113 ± 12.2, 59 **^****^** |
| K57/401E | 1.98 ± 0.0541, 54 **^****^** | 0.379 ± 0.0253, 198 **^****^** | 52.1 ± 2.01, 27 **^****^** |
| K57E | 3.08 ± 0.131, 85 **^****^** | 0.288 ± 0.0123, 166 **^****^** | 96.8 ± 5.79, 51 **^****^** |
| K401E | 3.08 ± 0.164, 85 **^****^** | 0.306 ± 0.0133, 174 **^****^** | 92.4 ± 6.28, 49 **^****^** |

**^a^** Relative to wild type

**Supplementary Table 5.** Apparent steady-state kinetic parameters at the physiological pH 7.4. Results are representative of the mean ± S.D. of three experiments. Data are also presented as the percentage difference of the mean compared to wild type (*WT*).

| **PDI** | ***k*_cat_ (×10^-2^ s^-1^, %^a^)** | **K_M_ (μM, %^a^)** | ***k*_cat_/K_M_ (×10^3^ M^-1^ s^-1^, %^a^)** |
| --- | --- | --- | --- |
| *WT* | 3.47 ± 0.0435, 100 | 0.777 ± 0.0593, 100 | 44.6 ± 4.00, 100 |
| K57/401Q | 2.74 ± 0.344, 79 **^***^** | 1.83 ± 0.285, 235 **^***^** | 15.0 ± 0.590, 34 **^****^** |
| K57Q | 3.63 ± 0.129, 105 | 1.69 ± 0.264, 218 **^***^** | 21.5 ± 1.91, 48 **^****^** |
| K401Q | 4.03 ± 0.0955, 116 **^**^** | 1.65 ± 0.179, 212 **^**^** | 24.5 ± 1.43, 55 **^****^** |
| K57/401A | 2.26 ± 0.103, 65 **^****^** | 1.92 ± 0.252, 247 **^****^** | 11.8 ± 1.97, 26 **^****^** |
| K57A | 3.24 ± 0.103, 94 | 1.41 ± 0.288, 181 **^*^** | 23.0 ± 3.39, 52 **^****^** |
| K401A | 3.41 ± 0.104, 98 | 1.45 ± 0.0858, 186 **^*^** | 23.6 ± 0.876, 53 **^****^** |
| K57/401E | 1.68 ± 0.141, 48 **^****^** | 1.84 ± 0.256, 237 **^**^** | 9.12 ± 0.489, 20 **^****^** |
| K57E | 2.94 ± 0.284, 85 **^**^** | 1.66 ± 0.296, 214 **^**^** | 17.7 ± 1.56, 40 **^****^** |
| K401E | 3.23 ± 0.154, 93 | 1.33 ± 0.148, 171 **^*^** | 24.3 ± 1.56, 54 **^****^** |

**^a^** Relative to wild type

**Supplementary Table 6.** Apparent steady-state kinetic parameters at pH 8.0. Results are representative of the mean ± S.D. of three experiments. Data are also presented as the percentage difference of the mean compared to wild type (*WT*).

| **PDI** | ***k*_cat_ (×10^-2^ s^-1^, %^a^)** | **K_M_ (μM, %^a^)** | ***k*_cat_/K_M_ (×10^3^ M^-1^ s^-1^, %^a^)** |
| --- | --- | --- | --- |
| *WT* | 3.07 ± 0.396, 100 | 2.22 ± 0.271, 100 | 12.94 ± 0.695, 100 |
| K57/401Q | 1.17 ± 0.109, 38 **^****^** | 2.46 ± 0.156, 111 | 5.74 ± 0.483, 34 **^****^** |
| K57Q | 1.70 ± 0.0974, 56 **^****^** | 2.13 ± 0.0440, 96 | 8.37 ± 0.277, 58 **^****^** |
| K401Q | 2.20 ± 0.258, 72 **^**^** | 2.46 ± 0.245, 111 | 9.78 ± 0.737, 65 **^****^** |
| K57/401A | 1.17 ± 0.166, 38 **^****^** | 2.53 ± 0.188, 114 | 4.26 ± 1.07, 33 **^****^** |
| K57A | 1.35 ± 0.121, 44 **^****^** | 1.60 ± 0.0159, 72 | 8.44 ± 0.745, 61 **^****^** |
| K401A | 2.01 ± 0.256, 66 **^***^** | 2.11 ± 0.607, 95 | 9.45 ± 1.17, 69 **^****^** |
| K57/401E | 0.473 ± 0.0541, 15 **^****^** | 2.32 ± 0.264, 105 | 2.01 ± 0.763, 15 **^****^** |
| K57E | 1.20 ± 0.485, 39 **^****^** | 1.99 ± 0.325, 90 | 4.26 ± 0.983, 44 **^****^** |
| K401E | 1.99 ± 0.353, 65 **^***^** | 2.33 ± 0.164, 105 | 8.54 ± 1.10, 62 **^****^** |

**^a^** Relative to wild type


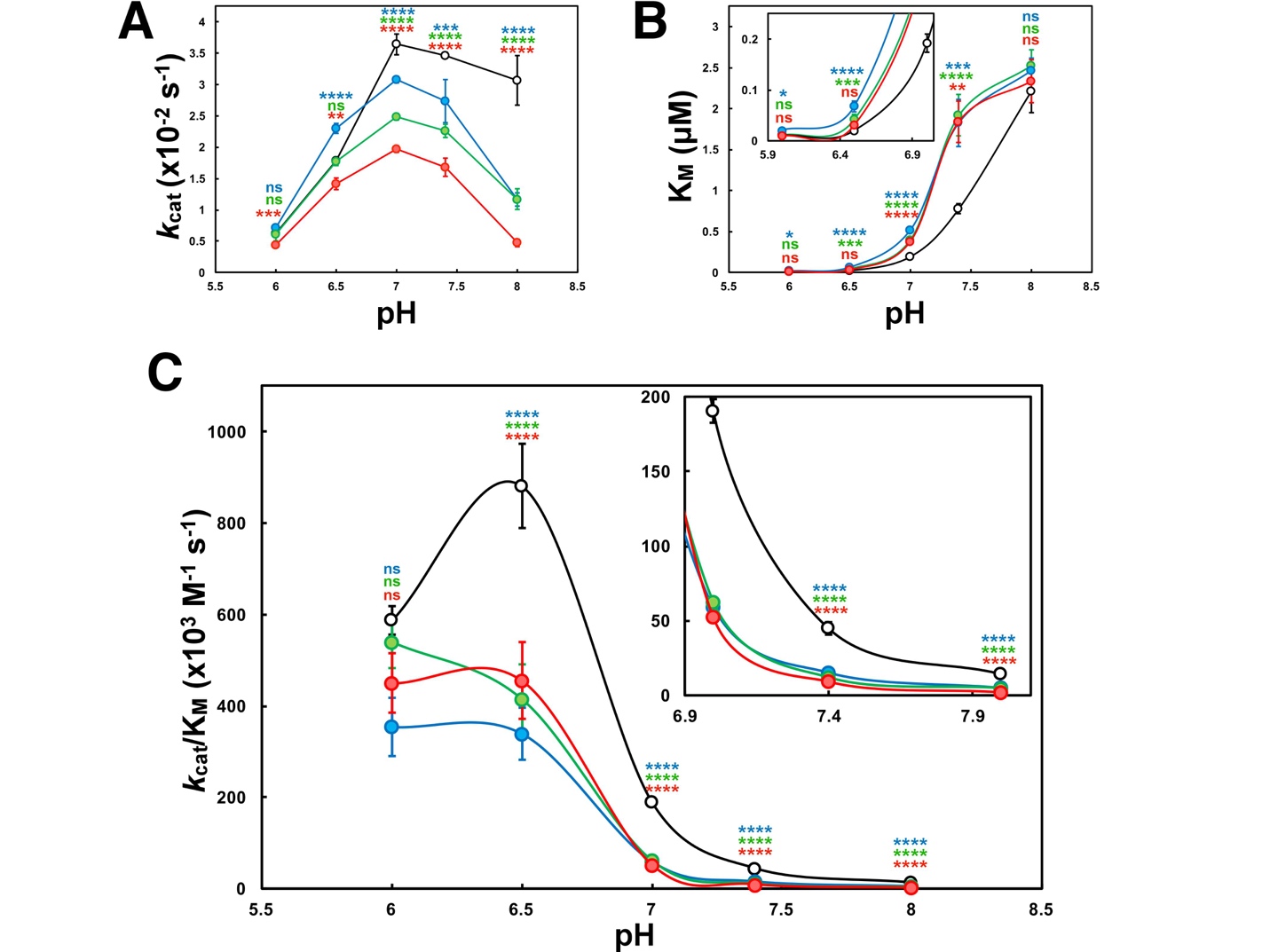


**Supplementary Figure 7.** Plots of the **(A)** *k*_cat_ and **(B)** K_M_ data from supplementary Tables 2-6: wild type (black circles); K57/401Q (blue circles); K57/401A (green circles); K57/401E (red circles).

*
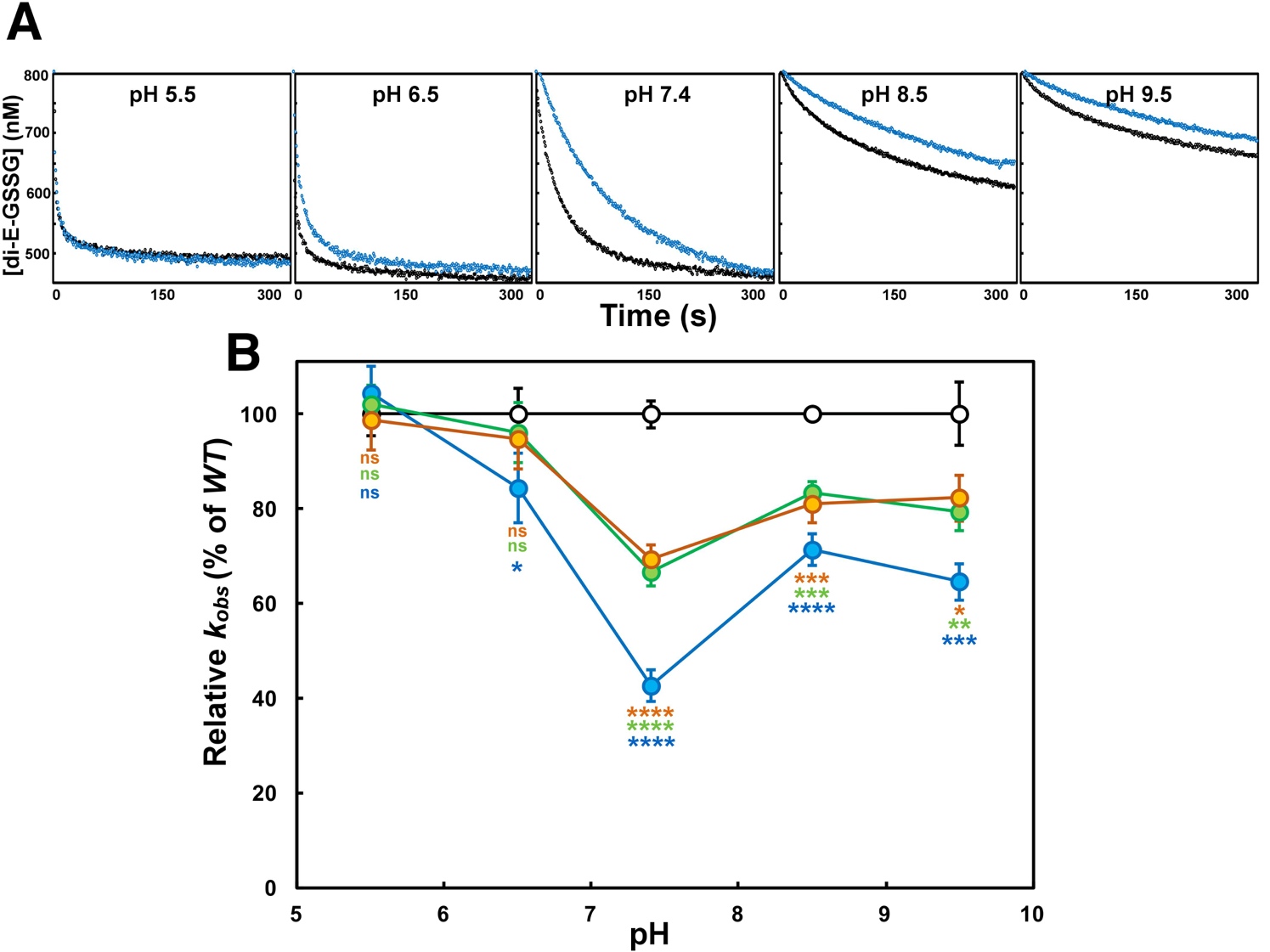
*

**Supplementary Figure 8.** Single turnover thiol-reductase activity of PDI. Representative data for the progress of the reduction of 1 μM di-E-GSSG by 80 nM wild type PDI (*WT*; black) and K57/401Q (blue) showing a single-phase reaction. Fluorescence data were related to the depletion of substrate (di-E- GSSG) over time. Rates become progressively slower with increasing pH due to the diminished nucleophilicity of Cys_N_ (pH >> p*K*_a_).

*
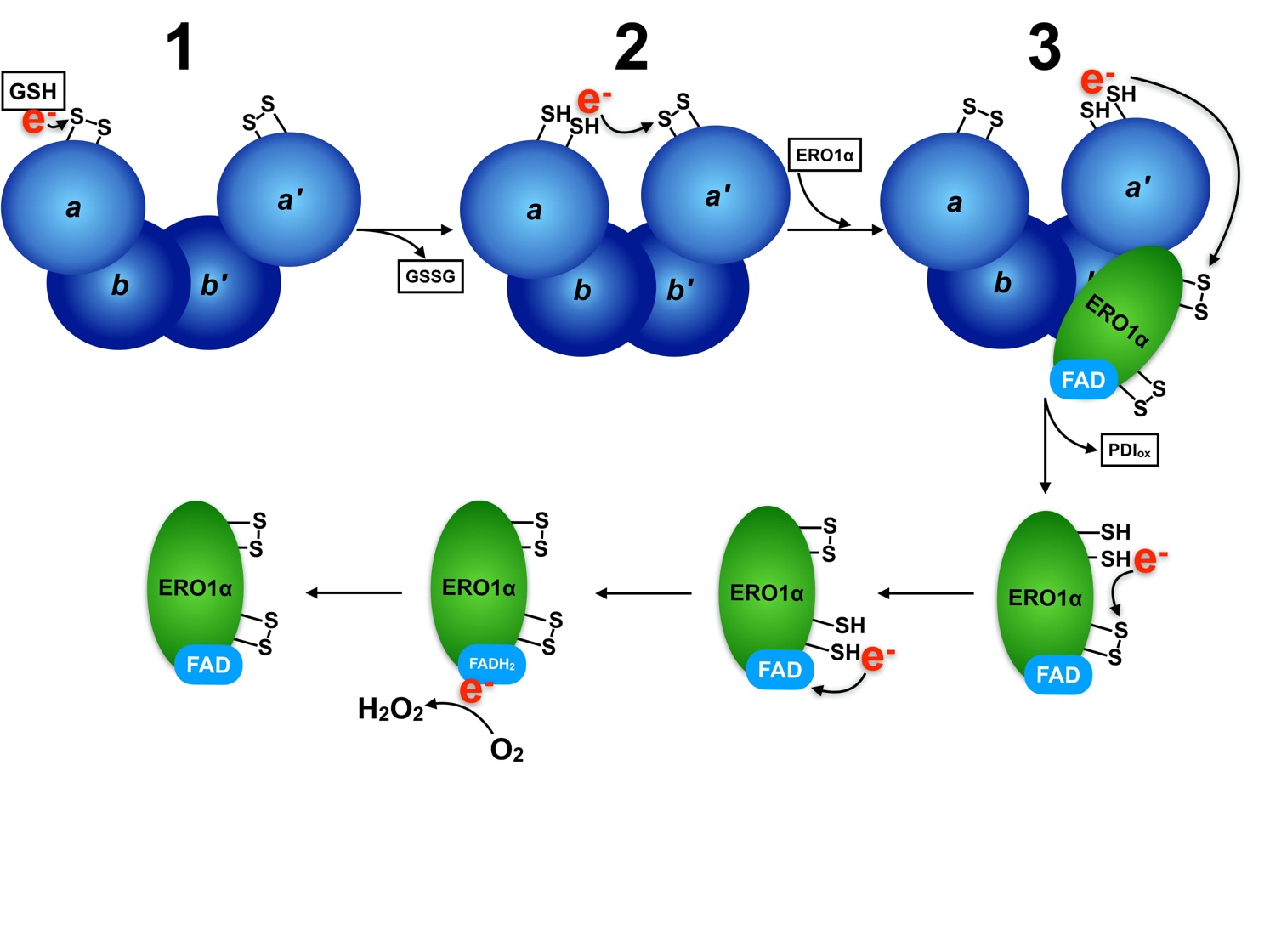
*

**Supplementary Figure 9.** Step-wise electron flow during oxidative protein folding mediated by the PDI-ERO1α interaction pathway of thiol-disulfide exchange. The *a*-domain of PDI oxidizes a reduced substrate (GSH; *step 1*), thereby becoming reduced (accepting two electrons denoted in red as *e*^-^). The *a'*-domain of PDI re-oxidizes the *a*-domain resulting in electron flow between PDI active sites (*step 2*). ERO1α binds the *b'*-domain of redPDI and selectively oxidizes the *a'*-domain by facilitating electron transfer to its regulatory loop disulfide (*step 3*). The electrons are subsequently transferred to the active site CxxC of ERO1α, which ultimately results in the reduction of FAD to FADH_2_. FADH_2_ reduces molecular oxygen (O_2_) to form hydrogen peroxide (H_2_O_2_)- regenerating oxidized ERO1α.


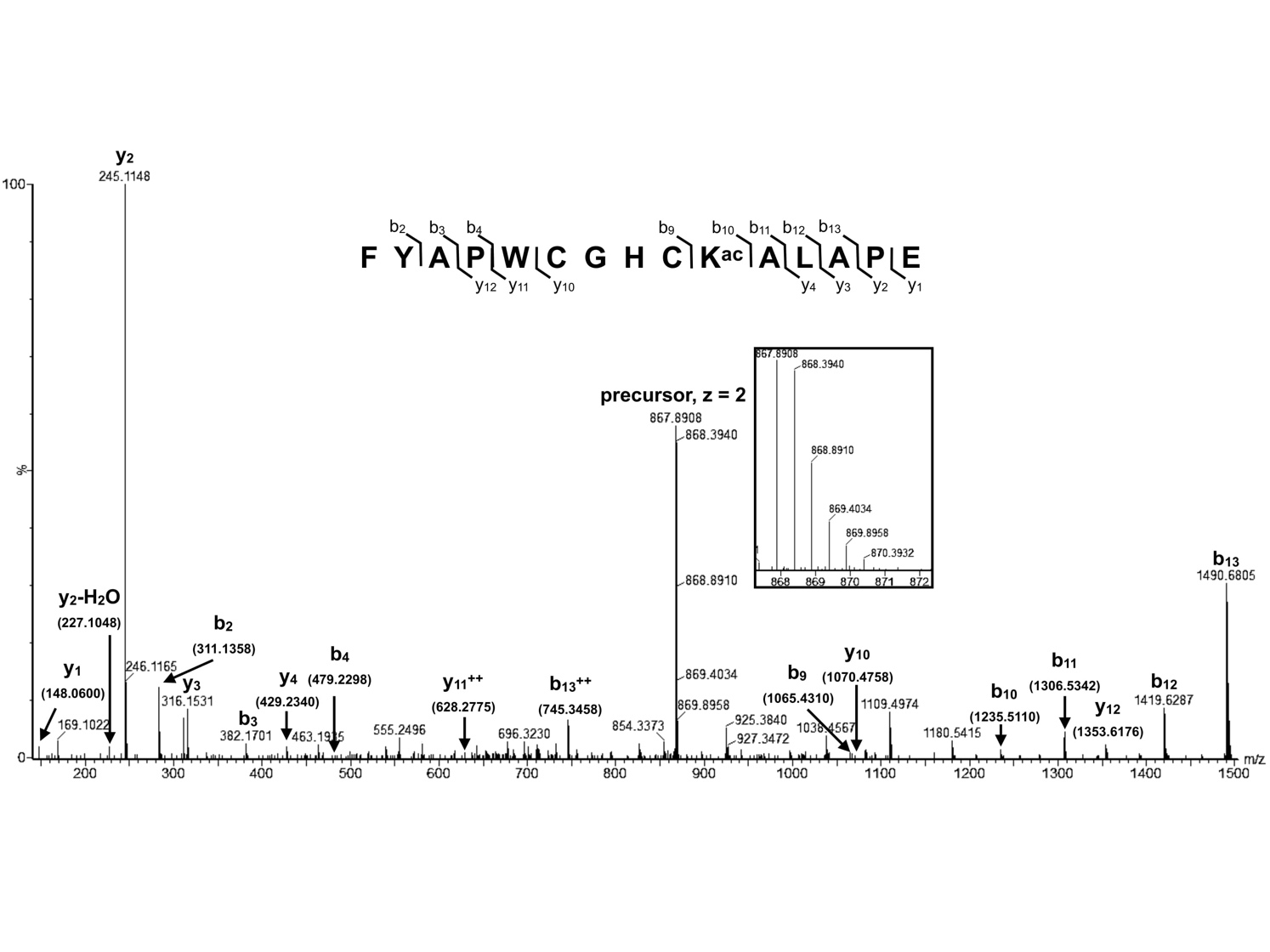


**A**


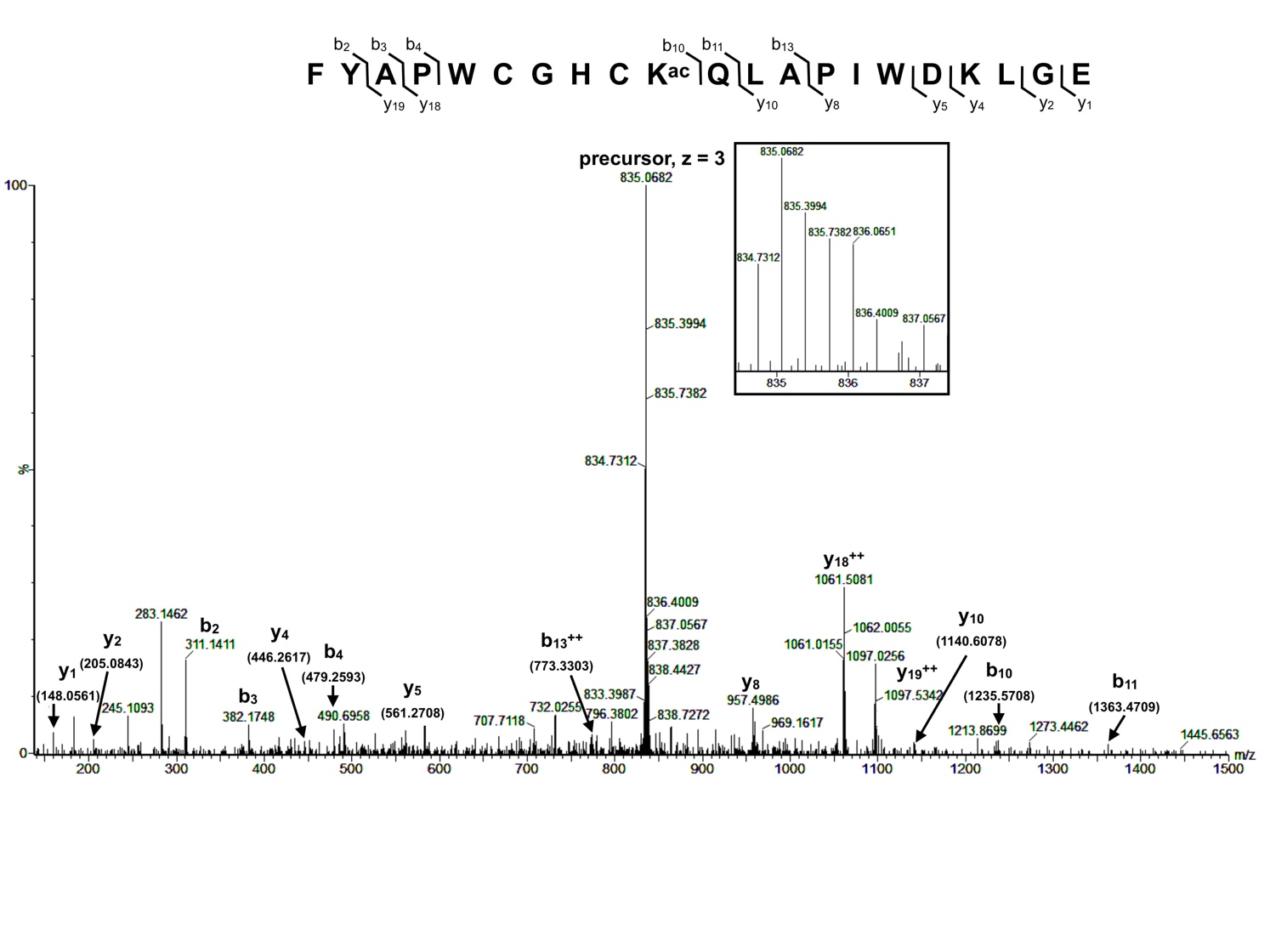


**B**

**Supplementary Figure 10.** Residues Lys^57^ of the *a*-domain and Lys^401^ of the *a'*-domain are acetylated by aspirin *in vitro*. Peptides from a GluC-digest of PDI underwent AuNP thiol-enrichment prior to performing UPLC-ESI MS/MS. **(A)** MS/MS spectrum of the PDI peptide (m/z 867.8908) containing acLys^57^. **(B)** MS/MS spectrum of the PDI peptide (m/z 834.7312) containing acLys^401^.


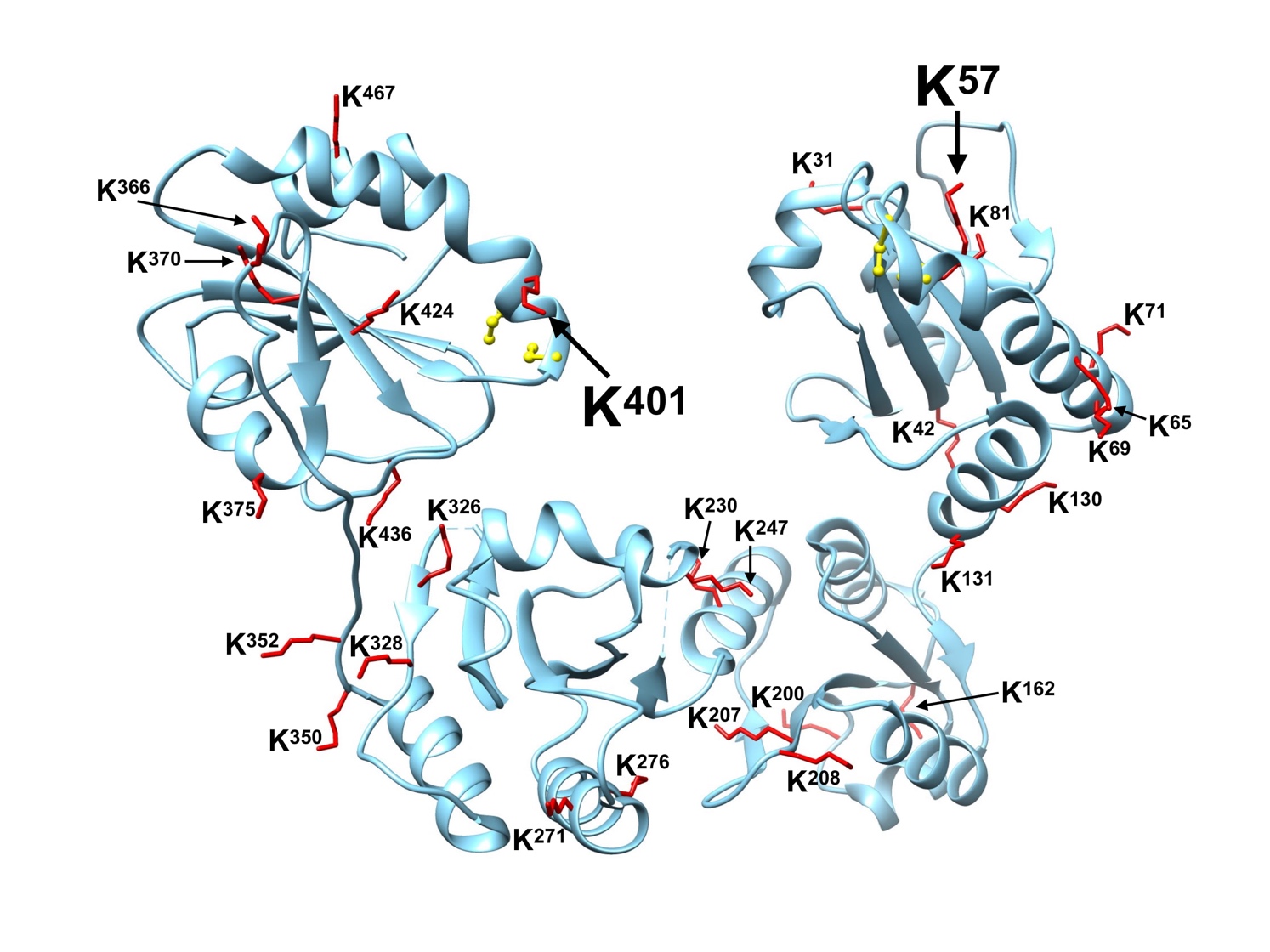


**Supplementary Figure 11.** A total of 28 acetyllysine residues were identified after treating PDI with ASA; mapped to the crystal structure of redPDI (PDB: 4EKZ (Wang et al., 2013)). Identification was confirmed by ProteinLynx Global Server search results of MS^e^ data (refer to the accompanying mass spectrometry supplementary spreadsheet). Those residues found to be acetylated are shown in red and labelled respectively. Hydrogens of the side chains have been omitted for clarity. The active site cysteines are depicted in yellow.

**Supplementary Table 7.** Kinetic parameters for untreated (control) and aspirin-acetylated (ASA) PDI enzymes. Data were extracted from **Figure 7**. Results shown represent the mean ± S.D. of three experiments.

| **PDI** | ***k*_cat_ (×10^-2^ s^-1^)** | **K_M_ (μM)** | ***k*_cat_/K_M_ (×10^3^ M^-1^ s^-1^)** |
| --- | --- | --- | --- |
| *WT*_control_ | 0.69 ± 0.03 | 1.27 ± 0.11 | 5400 ± 356 |
| *WT*_ASA_ | 0.31 ± 0.02 | 2.13 ± 0.07 | 1440 ± 396 |
| K57/401Q_control_ | 0.29 ± 0.05 | 2.51 ± 0.05 | 1140 ± 290 |
| K57/401Q_ASA_ | 0.13 ± 0.05 | 2.55 ± 0.06 | 517 ± 23 |

**Supplementary References:**

Faccenda, A., Bonham, C.A., Vacratsis, P.O., Zhang, X. & Mutus, B. (2010). Gold nanoparticle enrichment method for identifying S-nitrosylation and S-glutathionylation sites in proteins. J Am Chem Soc 132(33), 11392-11394. doi: 10.1021/ja103591v.

Freeman, R.G., Grabar, K.C., Allison, K.J., Bright, R.M., Davis, J.A., Guthrie, A.P., et al. (1995). Self-Assembled Metal Colloid Monolayers: An Approach to SERS Substrates. Science 267(5204), 1629-1632. doi: 10.1126/science.267.5204.1629.

Wang, C., Li, W., Ren, J., Fang, J., Ke, H., Gong, W., et al. (2013). Structural insights into the redox-regulated dynamic conformations of human protein disulfide isomerase. Antioxid Redox Signal 19(1), 36-45. doi: 10.1089/ars.2012.4630.
